# The vertical distribution and biological transport of marine microplastics across the epipelagic and mesopelagic water column

**DOI:** 10.1038/s41598-019-44117-2

**Published:** 2019-06-06

**Authors:** C. Anela Choy, Bruce H. Robison, Tyler O. Gagne, Benjamin Erwin, Evan Firl, Rolf U. Halden, J. Andrew Hamilton, Kakani Katija, Susan E. Lisin, Charles Rolsky, Kyle S. Van Houtan

**Affiliations:** 1 Monterey Bay Aquarium Research Institute, 7700 Sandholdt Road, Moss Landing, California 95039 USA; 2 Monterey Bay Aquarium, 886 Cannery Row, Monterey, California 93940 USA; 3 Arizona State University, Tempe, Arizona 85287 USA; 4 Nicholas School of the Environment, Duke University, PO Box 90328, Durham, NC 27708 USA; 5 Present Address: Scripps Institution of Oceanography, University of California San Diego, 9500 Gilman Drive, La Jolla, California 92093-0218 USA

**Keywords:** Marine biology, Ecosystem ecology, Environmental impact

## Abstract

Plastic waste has been documented in nearly all types of marine environments and has been found in species spanning all levels of marine food webs. Within these marine environments, deep pelagic waters encompass the largest ecosystems on Earth. We lack a comprehensive understanding of the concentrations, cycling, and fate of plastic waste in sub-surface waters, constraining our ability to implement effective, large-scale policy and conservation strategies. We used remotely operated vehicles and engineered purpose-built samplers to collect and examine the distribution of microplastics in the Monterey Bay pelagic ecosystem at water column depths ranging from 5 to 1000 m. Laser Raman spectroscopy was used to identify microplastic particles collected from throughout the deep pelagic water column, with the highest concentrations present at depths between 200 and 600 m. Examination of two abundant particle feeders in this ecosystem, pelagic red crabs (*Pleuroncodes planipes*) and giant larvaceans (*Bathochordaeus stygius*), showed that microplastic particles readily flow from the environment into coupled water column and seafloor food webs. Our findings suggest that one of the largest and currently underappreciated reservoirs of marine microplastics may be contained within the water column and animal communities of the deep sea.

## Introduction

The practical allure of plastic – a durable synthetic material that resists chemical and physical degradation – has translated to widespread environmental concern. Derived from petrochemicals, plastic was brought into large-scale global production during the mid-20^th^ century^1^. Plastic debris was first recorded at the surface of the Atlantic and Pacific Oceans in the early 1970s^2,3^. Given the continued, and now accelerating, large-scale production, use, and mismanaged disposal of plastic since that time, marine plastic pollution is now a significant environmental challenge spanning nearly all ecosystem types, and all levels of marine food webs^4^. Physical and chemical hazards related to entanglement in and ingestion of plastic debris across a diverse range of plastic sizes and types have generated broad ecological concerns^5–7^. Further, human health impacts stemming from both the chemical exposure to plastic debris from seafood consumption, as well as from toxins that adsorb onto plastic debris from the surrounding seawater, are currently unknown^8,9^.

Most global scientific studies describing the extent and amounts of oceanic marine plastic pollution have been confined to the surface layer of the ocean. However, deep pelagic waters within marine ecosystems dwarf all other available living space on Earth, and growing evidence demonstrates that plastic is accumulating within the animals, bottom sediments, and trenches of the deep sea^10–14^. Recent global inventories of floating plastic waste point to size-selective fragmentation and transport of microplastics to deeper waters through physical and biological processes^15,16^, as well as movement into marine food webs following trophic uptake (ingestion) and other physical processes (e.g., gill ventilation^17^), and passage through the food web^18–21^. To understand the distribution and accompanying ecological impacts of marine plastic pollution, deep water column measurements from ecologically important areas and from representative organisms within these communities are necessary.

**Figure 1:**
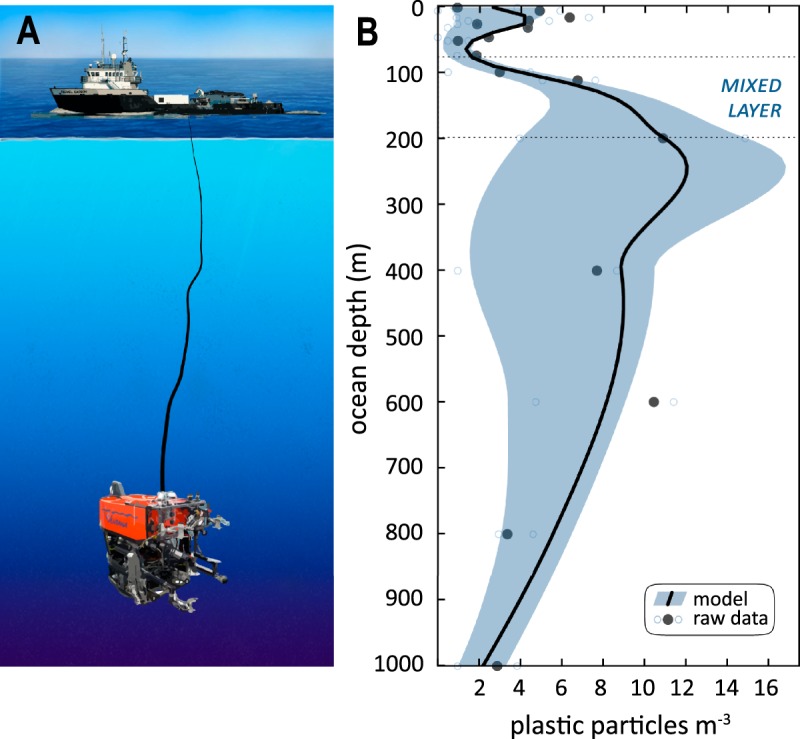
The highest concentration of ocean microplastics was between 200 and 600 m, in the offshore waters of the Monterey Bay pelagic ecosystem. (**A**) Sample collection schematic showing the ROV *Ventana* tethered to the R/V *Rachel Carson*, wherein ROV *Ventana* filtered seawater using purpose-built samplers across depths ranging from 5 to 1000 m. Seafloor depth at this sampling site is ~1,600 m. (**B**) Microplastic concentrations varied across sample depths and peaked just below the mixed layer (see SI). We observed the lowest concentrations at the ocean surface, yet these concentrations were comparable to the most extreme depths we sampled. Confidence intervals reflect the 90% quantile of the empirical distribution of Pearson correlation distances between the laser Raman spectra of degraded ocean plastic samples (fishing gear) and a spectral library of 14 pristine industrial plastic types (see SI).

Few previous studies have sampled and identified microplastics from sub-surface depths, using either multi-net trawls^22,23^ or water collected from seawater intake systems on underway research vessels (~3–11 m intake depth^24,25^). Additionally, microplastics have been analyzed from small volumes of discrete subsurface water samples collected with traditional CTD rosettes^2,26^. Given the reported range of dilute concentrations of marine microplastics collected with net tows at the sea surface^16,27^, daunting logistical constraints challenge the ability to make these same measurements in deep waters. In a first attempt to overcome these challenges, we modified *in situ* filtration equipment on deep-diving remotely operated vehicles (ROVs, Fig. 1A) and determined the concentrations of microplastics (>100 µm and <5 mm in size) in the deep midwaters of the ocean (Fig. 1B).

A diversity of fishes, crustaceans, cephalopods, and gelatinous animals inhabit the full water column of Monterey Bay, encompassing a suite of feeding interactions that fuel the populations of predatory commercial species^28^. Some of these forage species feed directly on particles of the same size range as microplastics, either by actively filtering and concentrating particles from the water column (filter feeders) or by selectively picking through individual particles (detritivores). Here, in conjunction with determining microplastic concentrations across epipelagic and mesopelagic depth zones, we examined the biological uptake of microplastic particles in two likely candidates of particle-feeding species, one filter feeder and one omnivore. Pelagic red crabs (*Pleuroncodes planipes*) are shallow-living, swimming squat lobsters with omnivorous diets, and are widely consumed by tuna, squid, sea birds, sharks, sea turtles, and marine mammals (e.g.^29,30^). Pelagic red crabs have both pelagic and benthic life stages and are generally distributed in the Eastern Tropical Pacific Ocean, but they can also be episodically abundant in Monterey Bay during warmer periods. Giant larvaceans (*Bathochordaeus stygius*, *B*. *mcnutti* and *B*. *charon*) are abundant filter-feeding appendicularians that graze on sinking and suspended particle fields using mucous mesh filters^31,32^. Giant larvaceans construct two nested feeding filters that are secreted as intricately structured mucopolysaccharides that can attain sizes greater than a meter in diameter. When clogged with particles from feeding, larvaceans discard these mucus feeding structures, which sink rapidly to the seafloor (“sinkers”) and deliver substantial amounts of carbon to the deep sea^33^. Using ROVs *in situ*, *B*. *stygius* has been documented filtering seeded microplastic particles from the water column, ranging from 10 to 600 µm in diameter, followed by ingestion and passage into fecal pellets^34^.

Within Monterey Bay, an iconic and ecologically-important deep submarine canyon ecosystem of the California Current, our efforts demonstrated that microplastic particles were widespread throughout the water column depth range we sampled (epipelagic and mesopelagic zones). Microplastic particles were directly taken up by key particle-feeding animals (giant larvaceans and pelagic red crabs), who removed microplastics from different depths within the water column and transported the material to surface, water column, and deep-seafloor food webs. Plastic polymer compositions were found to be similar to those reported in other published accounts (e.g.^2,25,35^), yet dissimilar from those in regional fishing gear we analyzed.

## Materials and Methods

### Study site and sample collection

To assess the vertical distribution and concentration of microplastic particles, high volumes of seawater were filtered *in situ* at discrete depths from the greater Monterey Bay pelagic ecosystem off the central California coast. A series of remotely operated vehicle (ROV) dives were conducted with the ROV *Ventana*, in April 2017 on the R/V *Rachel Carson* (see Fig. 1A). Two collection sites were chosen based on: (i) their proximity to outflow sources on land, and (ii) bottom depth within the submarine canyon (see SI for detailed map). The nearshore site, closest to potential land-based waste sources, was located at the mouth of Moss Landing Harbor (36.8°N, 121.82°W), where seawater collections reflect drainage from the Elkhorn Slough and surrounding agricultural and residential areas. The second, offshore site, where the majority of seawater samples were filtered for microplastic particles is a time-series site continuously visited since 1989, located approximately 25 km offshore in 1600 m of water (36.7°N, 122.05°W). Single ROV dives resulted in 1–2 depth-discrete samples filtered onto sterile nylon mesh (100 µm mesh) (see SI for sampler configuration, implementation, and limitations). However, given (i) the low sample sizes typical of deep-sea research, and (ii) our overarching objective to quantify microplastic concentrations across the water column, we combined concentration measurements for similar sampling depths across the two sites.

Water column samples were carefully collected over a total of three ship days (42 working hours) and ten individual ROV dives. The following selected depths were sampled for microplastic particles: 5 m (n = 3), 25 m (n = 5), 50 m (n = 2), 75 m (n = 1), 100 m (n = 1), 200 m (n = 1), 400 m (n = 1), 600 m (n = 1), 800 m (n = 1), 1000 m (n = 1). This overall depth range was selected to encompass full sampling across epipelagic (~0–200 m) and mesopelagic (~200–1000 m) zones. Discrete depths were chosen in a manner that balanced sampling effort (individual ROV dives) with even sampling across depths, while also targeting finer sampling resolution in the physically and biologically dynamic epipelagic zone. One of the 25 m samples was discarded due to challenges with flow and pressure. One additional ROV collection sampled water depths obliquely from 25 to 200 m; we used the median value of 112.5 m for this sample. Water samples were filtered *in situ* by purpose-built samplers and coupled pumps on the ROV, wherein the ROV moved forward at a select depth during sample collection as particles larger than 100-µm were collected onto sterile mesh. An integrated flowmeter recorded the volume of water filtered from each discrete depth. Filtered volumes of seawater ranged from 1,007 to 2,378 m^3^ per depth horizon. Field-blanks mirroring the exact avenues of the water sample collection process at depth were not feasible, but strict measures were taken to minimize contamination (details provided in SI).

We collected discarded giant larvacean particle-filtering houses (*Bathochordaeus* spp.) known as “sinkers” from discrete depths using detritus samplers on ROVs *Ventana* and *Doc Ricketts* after Robison *et al.*^33^. Briefly, we collected eight individual sinkers across a range of depths (251 to 2,967 m), aiming to both encompass and exceed the depth range of our water samples during January, February, and April of 2017. After ROV retrieval, sinker samples were filtered onto sterile mesh with a vacuum pump system within a controlled shipboard environment (sealed cold room with an isolated ventilation system). Sinker material was pulled onto glass fiber filters for subsequent Raman analysis to quantify microplastic particles and associated material composition.

Toward the end of the 2014–2016 El Niño event (September 2016), we collected beach-cast pelagic red crabs (*Pleuroncodes planipes*) from two proximate locations in Monterey, California. We randomly selected freshly-dead crabs, collecting the maximum permitted amount per location (*n* = 35), and preserved specimens at 0 °C. For a random subset of these samples, we measured basic morphometrics (carapace length, carapace width) and recorded the whole-body mass and removed the gastrointestinal tracts with solvent-cleaned dissection tools in a controlled laboratory environment (see SI for further details). A total of 24 individuals were examined to quantify microplastic ingestion and associated material composition.

### Microscopy and raman spectroscopy analysis

Isolated filters of seawater and animal-based samples were first visually assessed for potential plastic particles with a digital microscope alongside a stereo microscope. Morphological characters used to identify potential plastic particles were primarily color and shape. Particles were organized into gridded containers for Raman spectral analysis. Micro-Raman imaging was conducted using a Renishaw InVia confocal microscope and Raman spectrometer at magnifications consistent with 5x, 20x, 50x and 100x, and a numerical aperture of 0.75. Samples were analyzed using a 15 mW laser of a 488 nm wavelength at 5–10% laser intensity with exposure times of 10 seconds. Raman spectra were generated for particles of interest following individual particle extraction from seawater filters, sinker, and crab gut samples (see SI for more information). Each individual particle of interest (i.e., appeared to be a potential microplastic particle) was analyzed for Raman spectroscopy, and all data are reported here.

Multiple measures were taken to preclude microplastic particle contamination from the laboratory environment and the analysts. Prior to commencing analysis, individual filters were linked to control containers which were visually assessed for potential contamination at regular intervals during the experiment. The gridded tape holding particles of interest was kept covered as much as possible, and the composition and organization of individual particles were carefully tracked. Additionally, non-plastic clothing was worn during the analyses, and latex gloves were worn at all times.

### Data analysis and plastic composition identification

Raman spectra were generated for 14 plastic polymer types commonly identified from the marine environment. We used industrial-sourced virgin plastic materials to build a reference library of 12 Raman spectra, to which we sourced an additional two published spectra (see SI). We also sampled a variety of weathered plastic materials typical of local fisheries and boat operations in Monterey Bay and included them in the reference library. Selection was based on capturing a variety of renderings, colors, forms, and known applications in order to represent a broad diversity of local maritime activities, and also included materials from the ROV sampler (see Fig. S8 and Table S2 in SI).

Raman spectra ranged from 780 to 1750 cm^−1^ at resolution of 1 cm^−1^ which concurs with other studies successfully identifying polymers using Raman spectroscopy^36^. There were three static runs for each sample ranging from 520–2520 cm^−1^. Background noise and phase shifts between runs prevented the creation of a single, stitched, long-form spectrum. Thus, the center static run (1520 cm^−1^) was selected and a 15 cm^−1^ median window filter was applied to remove spurious cosmic ray detections^37^. Elevated intensity baselines due to fluorescence in spectra were corrected using a 7^th^ order polynomial baseline^38^. All spectra were then standardized with standard normal variate correction and min-max standardization, within 0–1^39^. Baseline correction and standardization were completed with the functions contained in the R packages ‘hyperSpec’ and ‘prospectR’, respectively^40,41^.

Unlabeled Raman spectra were compared against known reference polymer spectra with product moment correlation coefficients between all combinations of reference spectra to unspecified (water, crab, larvacean sinkers, and fishing gear) sample spectra^42^. This approach quantifies a measure of similarity often referred to as a Hit Quality Index^43^, analogous to a Pearson distance. With a matrix of coefficients for each sampled specimen relative to each reference polymer spectrum, the polymer assignment for each sample was the most closely correlated reference polymer. Given the often highly-degraded nature of marine microplastics, and the uncertainty that all sample particles were in fact plastic polymers, we developed a quantile-based cutoff for material assignments from the empirical distributions of Pearson distances in the fishing gear samples. The collected fishery samples are known plastic polymers that have also been weathered through exposure in the marine environment. The resulting distribution of Pearson distances between the fishing gear samples and the closest matching reference polymers (range: 0.05–0.53, 5% = 0.13, 50% = 0.22, 95% = 0.4) serve as a useful reference of Pearson distances for our remaining unassigned sample spectra. We subsequently used the Pearson distances at the reference quantiles throughout our analyses to frame uncertainty in our calculations. Given that our fishery samples were plastic polymers, but some had low similarities to the reference spectra (Pearson distances <0.1), we deemed unassigned spectra above the 5% Pearson quantile to be plastic polymers, but considered the 50% quantile (median) more suitable for definitive polymer assignments.

## Results and Discussion

### Water column microplastic concentrations

A total volume of 26,239 L of seawater from depths spanning 5 to 1000 m was sampled and examined for microplastic particles. Microplastic concentrations were highest in water samples collected from depths just below the mixed layer (15 particles m^−3^ at 200 m, Fig. 1B), at a deep site located 25 km from the nearest land. Microplastic concentrations near the sea surface (5 m) were among the lowest we measured (median 2.9 particles L^−1^), and were roughly equivalent to those of the deepest waters we sampled (1000 m, median 2.9 particles L^−1^). Concentrations were highest at intermediate depths into the mesopelagic zone. Although this study was not designed to determine concentration differences between nearshore and offshore locations, in the few cases where overlapping depth horizons were sampled at both locations (5 to 50 m depths), microplastic concentrations were higher at the offshore location (Fig. S6). Microplastic particles may thus be transported into the open-facing Monterey Bay ecosystem, by seasonally-distinct wind forcing and upwelling dynamics as a part of the greater California Current oceanographic system^44^.

In addition to identifying plastic particles at all ocean depths sampled, plastics were present in all pelagic red crab and giant larvacean sinker samples examined. The number of microplastic particles contained within a sinker ranged from 3 to 17 (mean 10.7 microplastic particles ± 5.3 particles standard deviation). While the gastrointestinal tracts of all pelagic red crab samples examined contained microplastic particles, the numbers of microplastic particles varied widely across individuals. Nearly half of the pelagic red crab samples (n = 11) contained fewer than 5 particles per individual, but three individual crabs contained greater than 10 microplastic particles each (see Fig. S12 for additional details).

Larvacean sinkers were collected by ROVs across depths ranging from 251 to 2,967 m, and individual sinkers containing the highest number of microplastic particles coincided with the depths of peak water column microplastic concentrations (Fig. S15). Pelagic red crabs were locally collected during mass beach strandings associated with the 2014–2016 El Niño Southern Oscillation event, and as a result we cannot resolve the precise depths or areas where the crabs we sampled had foraged. However, based on 30 years of ROV surveys in Monterey Bay^45^, the offshore depths where pelagic red crabs (100–150 m) and giant larvacean sinkers (200–250 m) are most abundant overlap with depths of peak plastic concentrations (Fig. 1B).

Our results corroborate previous *in situ* experiments demonstrating that giant larvaceans are capable of filtering microplastics from surrounding seawater^34^, with filtration rates significant enough (average 42.9 L hr^−1^) to bioconcentrate large amounts of environmental microplastics^46^. Larvacean sinkers thus function as transport vectors that deliver carbon alongside microplastic debris from shallower depths down to the seafloor^33^. Despite high abundances in the southern California Current^47^ and their importance as prey for animals at higher trophic levels, little is known about the feeding habits and filtration rates of pelagic red crabs. The pelagic red crabs in this study contained substantial numbers of microplastic particles in their gastrointestinal tracts (median 5, range 1–14 particles). Based on these data, the known depth preferences of pelagic red crabs, and observed water column concentrations of microplastics (Fig. 1B), we deduce a preliminary search volume rate sensu Bailey *et al.*^48^ of ~42.3 L hr^−1^ likely needed to obtain those plastic particles (see Fig. S12). Future *in situ* experiments, however, are needed to resolve the foraging behaviors and feeding rates of pelagic red crabs.

### Material composition and decomposition

**Figure 2:**
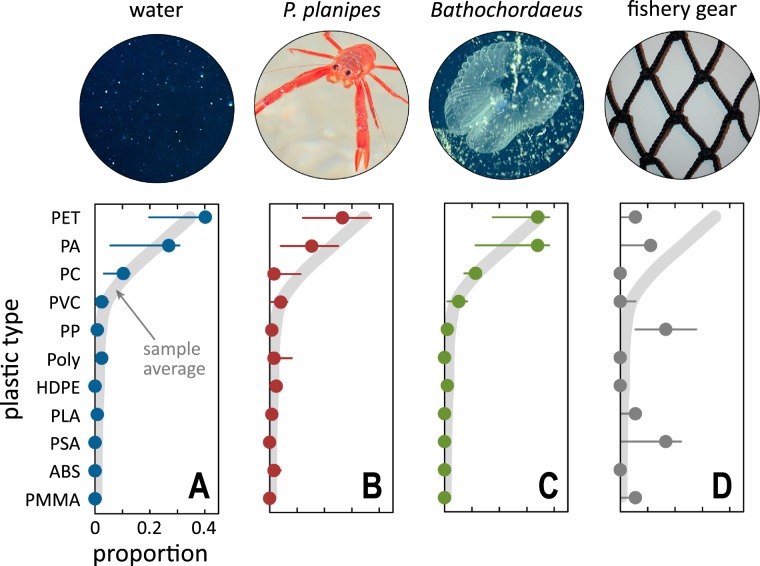
Polyethylene (PET) and polyamide (PA) were the dominant microplastics found in the water column and in particle-feeding marine life. Dominant plastic types identified from (**A**) water samples from 5 to 1000 m depths, (**B**) gastrointestinal tracts of pelagic red crabs (*Pleuroncodes planipes*), (**C**) discarded houses (“sinkers”) of giant larvaceans (*Bathochordaeus* spp.), and (**D**) representative materials from Monterey Bay fishery and maritime operations. Relative proportions are from individual sample sets; filled circles are the median, error bars represent the 90% Pearson quantiles. The thick gray trend line is the average microplastic proportional composition across water column and marine life samples (**A**–**C**), and is replicated across all panels. Unlike the water column and marine life samples, fishing gear samples were composed mostly of polypropylene (PP) and polystyrene acrylonitrile (PSA) materials. Tables S1 and S2 provide more details on the polymers.

Polyethylene terephthalate (PET) was the most common plastic identified from all depths of the water column samples (both nearshore and offshore sites, Fig. 2A), from the gastrointestinal tracts of pelagic red crabs (Fig. 2B), and from discarded larvacean sinkers (Fig. 2C). Polyamide (PA) was the second most common plastic polymer identified from the three sample types, followed by polycarbonate (PC) and polyvinylchloride (PVC). PET has a higher density than seawater and is a common material for single-use beverage bottles and packaging, while PA has a slightly lower density than PET and is used to make textiles and in the automotive industry (see Table S2 for other uses).

Taken together, the compositions of plastics sampled from the water column, pelagic red crabs, and larvacean sinkers (gray line, Fig. 2A–C) demonstrate that these two particle-feeding species may likely be taking up microplastic particles directly from the water column. The plastic composition of these samples, however, did not match the composition of representative fishing gear collected from Monterey Bay (Fig. 2D). By contrast, the fishing gear samples were primarily composed of polypropylene (PP) and polystyrene acrylonitrile (PSA, or acrylic, Fig. 2C). A spectral taxonomy of all plastic polymers shows the proximities and polymer relationships of all the polymers we assessed (Fig. S111). Thus, regional fishing gear was not likely a significant source for the microplastic particles sampled from across the water column, nor from the two animal samples.

Our findings detailing the prevalence of PET and PA in Monterey Bay are in agreement with results from other marine ecosystems^24,25^. While PC and PVC have been identified from marine waters and seafloor sediments^13,24,49^, they generally represent some of the smaller proportions of recovered marine plastics. Although readily distinguishable as pristine industrial products, environmental weathering may mute polymer spectra (see below) of ocean microplastic. Therefore, similar materials (e.g., PC and PET) may not be diagnostically distinguished after extensive exposure in marine systems.

Raman spectroscopy revealed that microplastics recovered from the Monterey Bay water column and marine life may be degraded in comparison to pristine industrial materials (Figs S6, S9). Commonly, plastic reference libraries for Raman spectroscopy are manually constructed by scanning known polymers^24,50^, or consist of large, proprietary commercial libraries only accessible through paid subscription. Here, Pearson correlations reflect the similarity of the Raman spectra of a sample particle suspected to be microplastic to known plastic polymers in our Raman spectra reference library. Because Pearson values decrease as samples become increasingly dissimilar to known polymers, and our reference library contains the most common types of polymers found in the ocean, we consider this statistic a potential proxy for material alteration or weathering (see SI for details).

The most frequent polymer assignments of our samples – PET and PA – had the highest Pearson correlations, or our highest confidence in those material assignments (Fig. S10). Moreover, microplastic particles from the pelagic red crab gastrointestinal tracts appeared to be significantly more altered than water column particles and by comparison to plastic from the larvacean sinker samples (Fig. S13). Giant larvaceans collect and filter microplastic particles from the water column by pumping water through filters, in a manner similar to the filtration methods employed with the ROV sampling^34^. This may be reflected in similar, and higher Pearson correlation distances, than the pelagic red crab samples. Digestive weathering within a crab’s stomach, though presently not well understood, is a potential source of plastic degradation, and may explain these patterns. Alternatively, though we did not visually observe this, biological material within crab intestinal tracts might have fouled the particle surface and altered the Raman spectra. A deeper understanding of the residence times and mechanisms surrounding the degradation of marine microplastics will be improved by large, open-access Raman reference libraries coupled with the development of automated polymer assignment methods, advancing the techniques presented here.

### Surface and deep-ocean food web cycling of microplastics

The movement of plastic waste from its production and usage on land to the surface layer of the global ocean is relatively well known^1,4,44^. Although multiple independent lines of evidence point to the accumulation and cycling of plastic waste in the waters and animal communities beneath the surface ocean e.g.^2,11–13,15,26^, the ecological and physical processes transporting plastic into the deep remain very poorly known. Here, alongside a detailed description of the vertical extent of the deep-water-column pool of microplastics, we document two distinct ecological pathways through which pelagic particle feeders transport microplastic to deeper waters, and ultimately to the seafloor.

**Figure 3:**
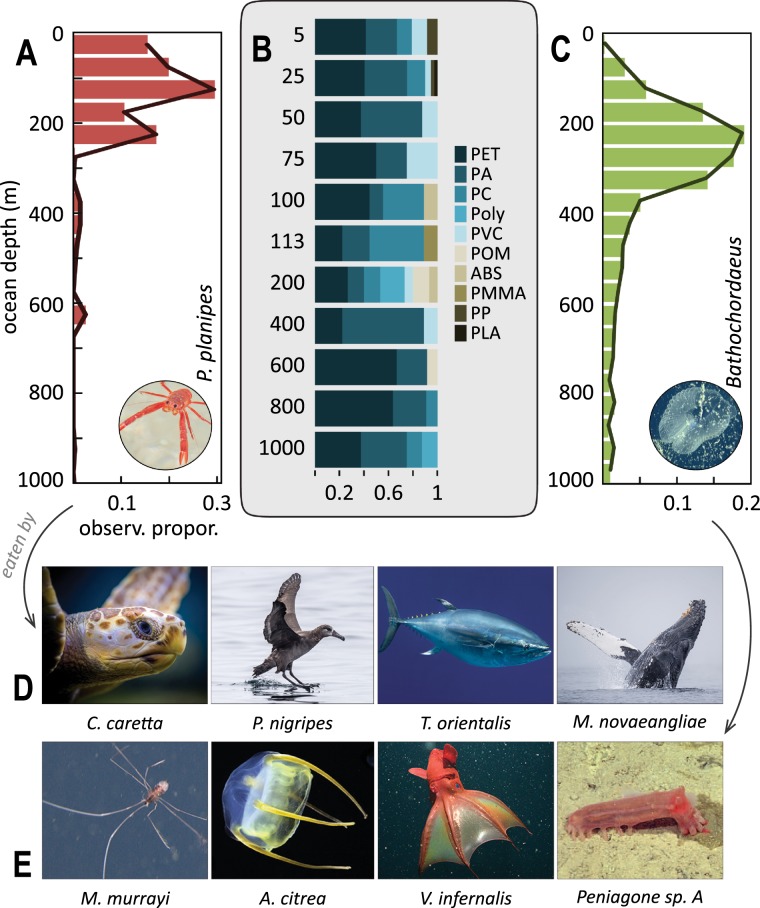
Ingested microplastics are transported into marine food webs by particle feeders. Depth distributions of (**A**) *Pleuroncodes planipes* and (**C**) *Bathochordaeus* sinkers from 30 years of ROV observations. Microplastic materials from (**B**) different depths of the water column are transported through the ocean food web by common (**D**) water column (*Caretta caretta*, *Phoebastria nigripes*, *Thunnus orientalis*, *Megaptera novaeangliae*) and (**E**) deep-sea (both pelagic and benthic) (*Munneurycope murrayi*, *Aegina citrea*, *Vampyroteuthis infernalis*, *Peniagone* sp.) organisms. While some plastic materials are widespread throughout the water column (PET, PA, PC, PVC), other materials appear to be restricted to the surface (PP, PLA) or sub-surface waters (P, POM, ABS, PMMA).

The depth distributions of giant larvaceans (the animals themselves and their discarded sinkers) and pelagic red crabs are well known within Monterey Bay (Fig. 3A,C). Pelagic red crabs are episodic components of the Monterey Bay pelagic ecosystem, as observed by multi-decadal ROV surveys^45^. We identified the peak of microplastic concentration (Fig. 1B) and the highest diversity of plastic material types (Fig. 3B) at the base of the sunlit epipelagic zone, where the water column transitions down into dimly lit mesopelagic depths. In addition to the pelagic red crabs (Fig. 3A) and larvaceans^46^ (and their discarded sinkers, Fig. 3C) this depth range encompasses a high density and diversity of important food web interactions^28^.

Altogether, our results reveal a dynamic and effective removal mechanism for transferring microplastics deep into the water column, and then into coupled pelagic and benthic food webs. Pelagic red crabs are widely fed upon by surface-dwelling and upper water column predators such as seabirds, sea turtles, marine mammals, and tunas (Fig. 3D; e.g.^29,30^). ROVs have documented discarded larvacean sinkers and larvaceans themselves, being eaten by a broad range of midwater and benthic species during and after their descent to the seafloor (Fig. 3E).

## Conclusions

This study provides direct evidence that a potentially large pool of marine microplastics may exist within the largest living space on Earth, the deep-sea water column. The Monterey Bay marine ecosystem is part of a network of marine protected areas and we found that water column microplastic concentrations match and exceed those found in other marine regions^16,24,25,51^. New monitoring and sampling technologies are necessary to access and inventory the full scale of microplastic pollution in the deep sea in a systematic and robust manner. Intensive sea-going surveys are logistically demanding and very costly, but increased technology investments are necessary for advancing our understanding of global marine ecosystems. Our results build upon previous work, suggesting that plastic pollution extends much further and more extensively into the waters, sediments, and animal communities of the deep sea^11,13,52^. As plastic waste generation is predicted to continue to grow for the rest of this century^49^, large-scale conservation and mitigation efforts must consider the enormous spatial (both horizontal, and vertical) and ecological scale of the problem that these new findings reveal.

## Supplementary information

Supplementary Information

## Data Availability

The datasets generated and analyzed during the current study are available in the Open Science Framework repository, https//osf.io/j6gmx/.
