# Author Correction: The vertical distribution and biological transport of marine microplastics across the epipelagic and mesopelagic water column

**DOI:** 10.1038/s41598-020-57573-y

**Published:** 2020-01-14

**Authors:** C. Anela Choy, Bruce H. Robison, Tyler O. Gagne, Benjamin Erwin, Evan Firl, Rolf U. Halden, J. Andrew Hamilton, Kakani Katija, Susan E. Lisin, Charles Rolsky, Kyle S. Van Houtan

**Affiliations:** 1 Monterey Bay Aquarium Research Institute, 7700 Sandholdt Road, Moss Landing, California, 95039 USA; 2 Monterey Bay Aquarium, 886 Cannery Row, Monterey, California, 93940 USA; 3 Arizona State University, Tempe, Arizona 85287 USA; 4 Nicholas School of the Environment, Duke University, PO Box 90328, Durham, NC 27708 USA; 5 Present Address: Scripps Institution of Oceanography, University of California San Diego, 9500 Gilman Drive, La Jolla, California, 92093-0218 USA

Correction to: *Scientific Reports* 10.1038/s41598-019-44117-2, published online 06 June 2019

This Article contains a typographical error in the Data Availability section where,

“The datasets generated and analyzed during the current study are available in the Open Science Framework repository, https//osf.io/j6gmx/.”

should read:

“The datasets generated and analyzed during the current study are available in the Open Science Framework repository, https://osf.io/j6gmx/”

