# Supplementary material for: The vertical distribution and biological transport of marine microplastics across the epipelagic and mesopelagic water column
Source: Sci Rep. 2019 Jun 6;9:7843. doi: 10.1038/s41598-019-44117-2 (PMC6554305; doi:10.1038/s41598-019-44117-2)
Supplement: Supplementary file 1 — Supplementary Information [file 41598_2019_44117_MOESM1_ESM.docx]

**ONLINE SUPPLEMENTARY MATERIAL**

“The vertical distribution and biological transport of marine microplastics across the epipelagic and mesopelagic water column”

by C. Anela Choy, Bruce H. Robison, Tyler O. Gagne, Benjamin Erwin, Evan Firl, Rolf U. Halden, J. Andrew Hamilton, Kakani Katija, Susan E. Lisin, Charles Rolsky, Kyle S. Van Houtan

This supplement presents additional information on methods, figures, and tables that provide added data and clarification. Analysis code and data can be found in the public repository: **osf.io/j6gmx/**

# Materials & Methods in Detail

## *Sample Collection*

### Water Column Microplastic Particles

Water samples were collected from the greater Monterey Bay pelagic ecosystem off the central California coast using a series of remotely operated vehicle (ROV) dives with the ROV *Ventana*, in April 2017 on the R/V *Rachel Carson* (see Fig. 1A). Two collection sites were chosen based on: (i) their proximity to outflow sources on land, and (ii) bottom depth within the submarine canyon (Fig. S2). The onshore site nearest to land-based waste sources was located at the head of Moss Landing Harbor (36.8°N, 121.82°W), where water collections reflect drainage from the Elkhorn Slough and greater area. The second offshore site is a continuously visited time-series site since 1989, Midwater 1 (36.7°N, 122.05°W), approximately 25 km offshore in 1600 m of water. To assess the vertical distribution and concentration of microplastic particles, discrete samples were taken at each site. A single ROV dive resulted in 1-2 discrete samples due to the sampler configuration (Fig. S3). At the nearshore site, five samples were taken from 5 m, two at 25 m, 50 m, and 75 m. From the offshore site, 11 samples were taken from 5 m, three at 25 m, 50 m, 100 m, 200 m, 400 m, 600 m, 800 m, and 1000 m. One of the two 25 m samples from the nearshore site was discarded due to flow and pressure sampling challenges. One additional collection sampled water depths obliquely from 25 to 200 m, we used the median value of 112.5 m for this sample.

To isolate potential microplastic particles from the water column, we developed a filtration system with modified 7.5 L collection buckets (“detritus samplers”, see *1*) and isolated filter rings outfitted with sterile 100-µm mesh. The filtration system was coupled to existing pumps on the ROV in such a manner that only the front or top-side of the filter was exposed to filtered seawater in the direction of collection. To the extent possible, minimized use of plastic materials by designing aluminum parts in the sampler. A flow meter was integrated into the system to record sample volume. At all depths, the ROV moved forward in the water column during collection to reduce the sample contact with materials from the ship or the ROV. We also undertook additional contamination prevention measures while sampling at sea, including not opening the sampler outside of a controlled container environment and quickly handling exposed samples while wearing cotton or wool clothing.

### Animal Samples

We collected discarded giant larvacean houses (*Bathochordaeus* spp.) known as “sinkers” using detritus samplers on ROVs *Ventana* and *Doc Ricketts* (see *2*). Briefly, “detritus samplers” were used to collect eight individual sinkers from depths ranging 251 to 2,967 m during January, February, and April of 2017. For a subset of sinker samples, a sample of ambient seawater was collected adjacent to the sinker to serve as a control sample. Using a vacuum pump system in a controlled shipboard environment (sealed cold room with an isolated ventilation system) we filtered sinker material onto glass fiber filters (EMD Millipore™, 4.7 cm diameter, 1.0 μm pore size) for subsequent Raman analysis.

Toward the end of the 2014-2016 El Niño event (September 2016), we collected beach-cast pelagic red crabs (*Pleuroncodes planipes*) from two proximate locations in Monterey, California. We selected freshly dead crabs, and preserved them at 0°C, for later analysis in a clean laboratory environment. To prevent contamination of samples, researchers wore protective clothing (Tychem® QC deluxe coveralls, nitrile gloves) during specimen preparations. We measured basic morphometrics (carapace length, carapace width) with carbon fiber calipers (Fisherbrand™ Traceable™) and recorded the whole-body mass on a microbalance.

With a scalpel, we made a longitudinal slit along dorsal carapace, revealing the entire gastrointestinal tract. Under a standard laboratory dissection microscope (10-25x power), we used stainless-steel probes and precision forceps to identify and extract potential microplastic particles (predominantly fibers and fragments of < 5 mm length) from the adjoining biological material (amorphous, soft, green-brown material that was easily separated). We placed particles of interest on glass fiber filters (EMD Millipore™, 4.7 cm diameter, 1.0 μm pore size) for subsequent Raman analysis. When simple extraction proved difficult (i.e. when the gastrointestinal tract was filled with large amounts of biological material) we removed all gut contents and placed them in a glass filtering stack. We then irrigated the samples with deionized water, while a vacuum pump spread the gut contents in a thin layer across glass fiber filters. We placed all glass filters in previously-rinsed, covered glass petri dishes. We wrapped petri dishes within combusted aluminum foil pouches, and stored them at 0°C.

## *Sample Processing and Raman Analysis*

Particles of interest were individually extracted from glass fiber filters, and then Raman spectra were generated for those particles and for the fishery gear samples, followed by a partial reference library of plastic polymer spectra.

Isolated filters of water and animal samples were visually assessed for potential plastic particles. Microscopic visual analysis was conducted with a digital microscope (Leica DM500 HD, no. EZ4W), alongside a stereo microscope (Olympus SZ51, no. SZ2-ILST). The stereo microscope magnified a gridded container enclosing tape to store and organize particles of interest. From each filter, the digital microscope was used to locate and carefully remove particles of interest, according to predefined visual cues for inorganic materials. Morphological characteristics such as color (bright colors, especially reds and blues) and shape (e.g., fragments and fibers) were the primary visual cues used. We were not conservative in selecting potential microplastic particles, choosing to also analyze particles for Raman that did not necessarily align with these visual cues. Particles were organized into the gridded containers for subsequent Raman spectral analysis.

Micro-Raman imaging was conducted using a Renishaw InVia confocal microscope and Raman spectrometer at magnifications consistent with 5x, 20x, 50x and 100x, and a numerical aperture of 0.75. Samples were analyzed using a 15 mW laser of a 488 nm wavelength at 5-10% laser intensity using exposure times of 10 sec. The calibration standard in all data collection was silicon (111) wafer material. Focusing was conducted incrementally using the 5x, 20x and finally, 50x lenses. We used three static wavelength runs of 520, 1520, and 2520 cm^-1^ (but see below) that enabled quick modifications to the spectrometer depending on particle degradation. Due to the highly-degraded nature of ocean microplastic particles, we frequently modified the laser intensity to gather clearer spectra.

Multiple measures were taken to minimize microplastic contamination from the laboratory environment and the analysts. Prior to beginning analysis, individual filters were linked to control containers which were visually assessed at regular intervals along the experiment. The gridded tape holding particles of interest was kept covered, and the composition and organization of individual particles was carefully tracked. Additionally, non-plastic clothing was worn during analysis, and latex gloves were worn at all times.

## *Data Analysis & Plastic Identifications*

### Plastic Types

Raman spectra were generated for 14 plastic polymer types commonly identified from the marine environment. We used industrial-sourced virgin plastic materials to build a reference library of 12 Raman spectra, to which we sourced an additional two published spectra (see Table S2, and *3*). Strict caution was taken to minimize potential environmental microplastic contaminants during field collections and laboratory processing. We also sampled a variety of weathered plastic materials typical of local fisheries and boat operations in Monterey Bay. Selection was based on capturing a variety of renderings, colors, forms, and known applications in order to represent a broad diversity of local maritime activities and included materials from the ROV sampler. Fig. S8 provides images of the selected samples, and Table S1 provides material descriptions, uses, and polymer assignments from Raman spectroscopy. These materials represent an array of plastic lines, nets, ropes, traps, and other types of fishery gear.

### Raman Spectra Preprocessing

All Raman spectra, unlabeled and known reference, ranged 780 to 1750 cm^-1^ at resolution of 1 cm^-1^ (Fig. S5). This range is in concurrence with other studies successfully identifying polymers using Raman spectroscopy (*4*). Though three static runs for each sample ranging from 520-2520 cm^-1^, background noise and phase shifts between runs prevented our ability to create a single, stitched, long-form spectra. We therefore selected the center static run (1520 cm^-1^) and ran a 15 cm^-1^ median window filter to remove spurious cosmic ray detections (*5*). Raman spectra of degraded samples had an amplified intensity level as a function of fluorescence in the sample. While the narrow peaks in Raman intensity at various wavenumbers represent the vibration of chemical bonds, the background fluorescence can make identification difficult due to the correlation between Raman shift and intensity (*6*). Elevated intensity baselines due to fluorescence in spectra were corrected using a 7^th^ order polynomial baseline (*6*). All spectra where then standardized with standard normal variate correction and min-max standardization, within 0-1 (*7*). Baseline correction and standardization was completed with the functions contained in the R packages `hyperSpec` and `prospectR`, respectively (*8, 9*).

### Raman Spectra Similarity

Unlabeled Raman spectra were compared against known reference polymer spectra with product moment correlation coefficients between all combinations of reference spectra to unknown (water, crab, larvacean sinkers, and fishery gear) sample spectra (*10*). This approach quantifies a measure of similarity often referred to as a Hit Quality Index (*11*), analogous to a Pearson distance. With a matrix of coefficients for each sampled specimen relative to each reference polymer spectra, the polymer assignment for each sample was the most closely correlated reference polymer. (Table S10 provides decays of product moment relative ranks for all assigned polymers.)

Given the highly-degraded nature of ocean microplastics, and the uncertainty that sample particles are in fact plastic polymers, we developed a quantile-based cutoff for material assignments from the empirical distributions of Pearson distances in the fishery gear samples. The fishery samples we collected have the unique property of being known to be composed of plastic polymers, but also have been weathered through exposure in the marine environment (Fig. S6). The resulting distribution of Pearson distances between the fishery gear samples and the closest matching reference polymers (range: 0.05-0.53, 5%=0.13, 50%=0.22, 95%=0.4) serve as a useful reference of Pearson distances for our remaining unassigned sample spectra. We subsequently used the Pearson distances at the reference quantiles throughout our analyses to frame uncertainty of our calculations. Given that our fishery samples were plastic polymers, but some had low similarities to the reference spectra (Pearson distances < 0.1), we deemed unassigned spectra above the 5% Pearson quantile to be plastic polymers, but considered the 50% quantile (median) more suitable for definitive polymer assignments.

All spectra preprocessing and identification script is available in the online repository.

**Figure S1. Hydrographic depth profiles of temperature, salinity, and oxygen data characterizing the “offshore” and “nearshore” sampling sites where microplastic particles were collected from the greater Monterey Bay submarine canyon ecosystem.** Depth is in meters, temperature in degrees Celsius, salinity in PSU, and oxygen in mL/L. These data in situ are used to characterize the mixed layer depth in Figure 1b in the main text. See Fig. S2 for geographic locations of offshore and nearshore sampling sites.

**Figure S2. Monterey Bay submarine canyon pelagic ecosystem.** Bathymetric map includes the two main sampling locations of the nearshore and offshore sites, where microplastics were collected throughout the water column. Discarded larvacean houses were collected primarily near the offshore site. The ocean-land boundary is demarcated by the black line, and a color scale is provided for elevation on land and bottom depth at sea (both in meters). At sea, darker blue colors indicate greater depths, revealing the offshore submarine canyon. The bottom depth at the offshore site is approximately 1,600 m, and 550 m at the nearshore site. Latitude and longitude decimal degree graticules are provided, with latitude in decimal degrees North and longitude in decimal degrees West (indicated by the negative sign).

**
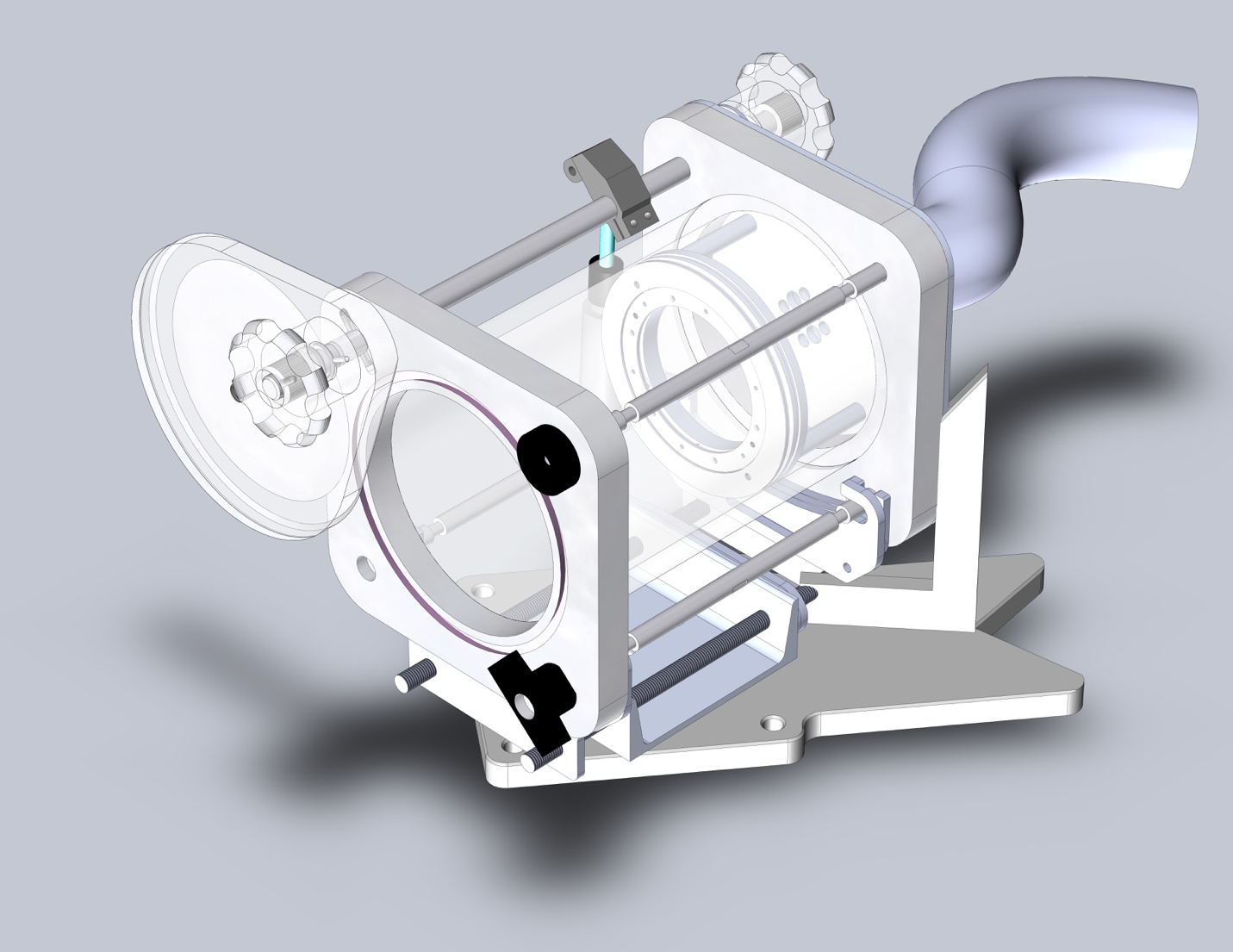
**

**Figure S3. Modified ROV sampler used to filter microplastic particles from the Monterey Bay deep water column.** The ROV would descend to a target sampling depth, we would open the sample door and move forward while filtering water across the filter, within a single depth horizon. Upon completion of sample collection, the water-tight sample door was closed, sealing off the water sample until recovery. Aluminum filter ring outfitted with sterile 100-µm mesh sits nested deep within the sampler, with the direction of flow retaining microplastic particles only on the top surface of the filter.

| **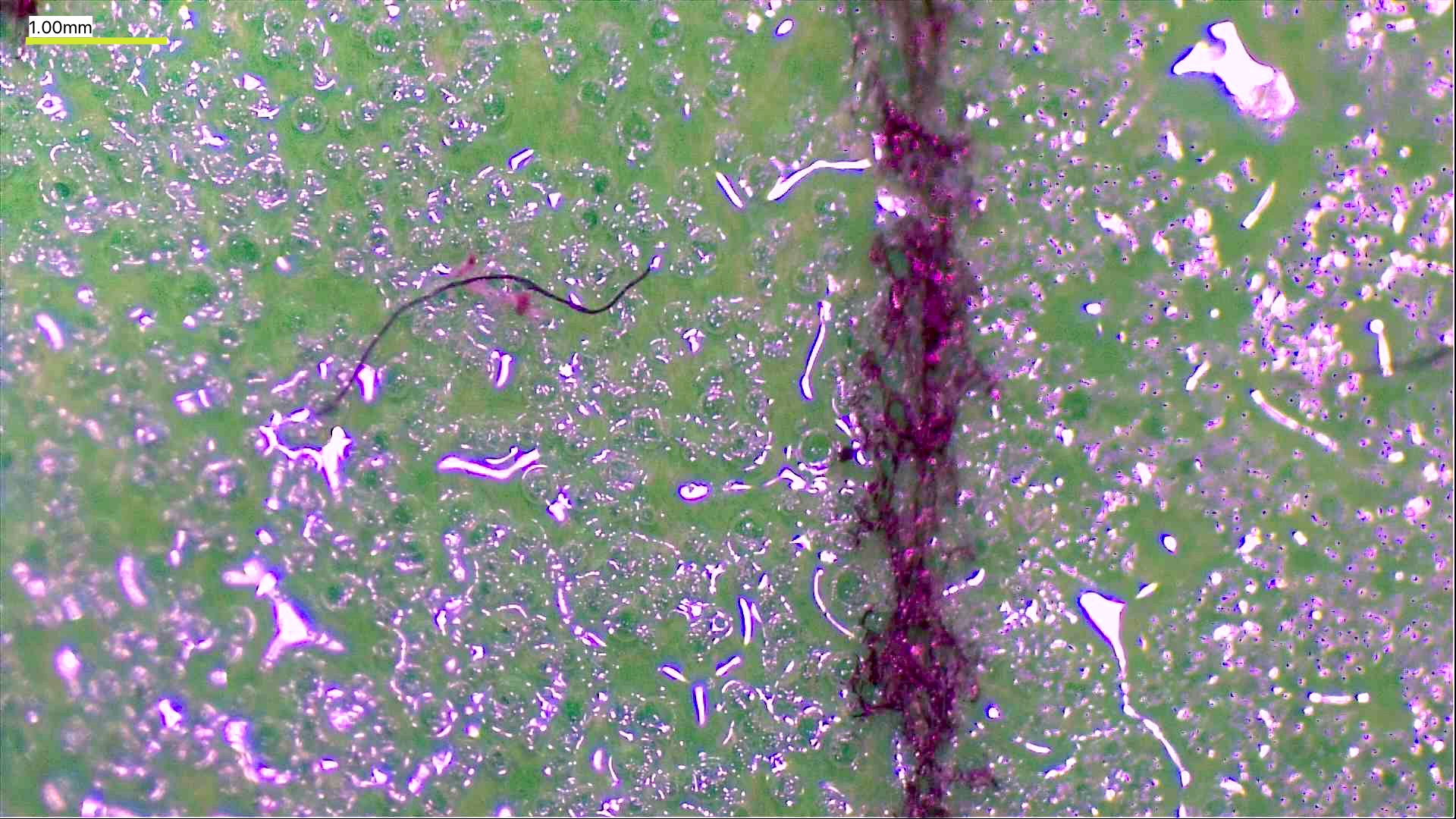** |
| --- |
| **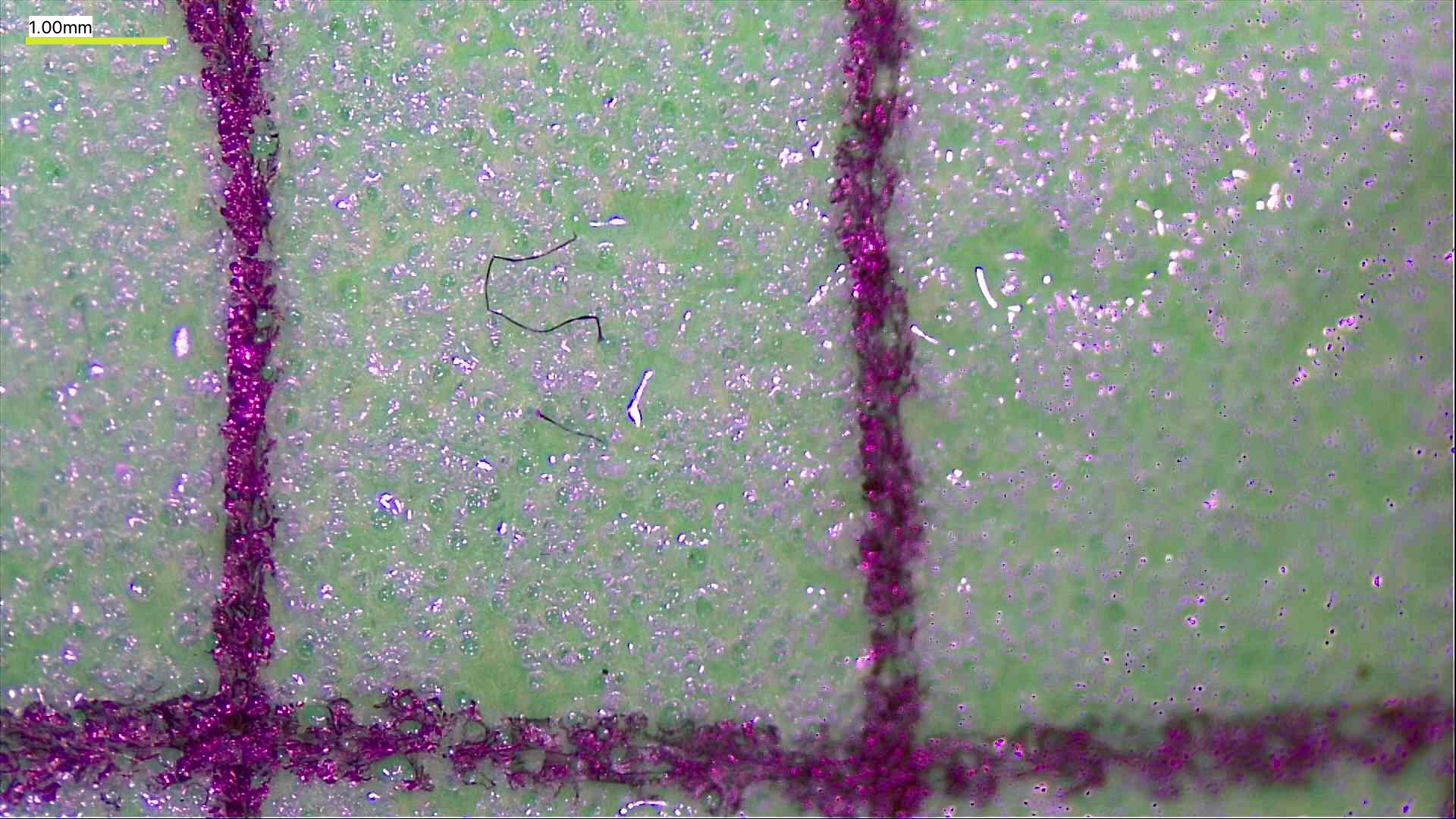** |

**Figure S4. Microplastic particles found in marine life samples from the Monterey Bay pelagic ecosystem.** The majority of microplastics identified consisted of highly degraded fibers similar to these pictured. Note the 1-mm scale bar shown (upper left) in yellow. The purple vertical line in the lower photo is part of a manual grid drawn to organize individual particles for subsequent Raman analysis.


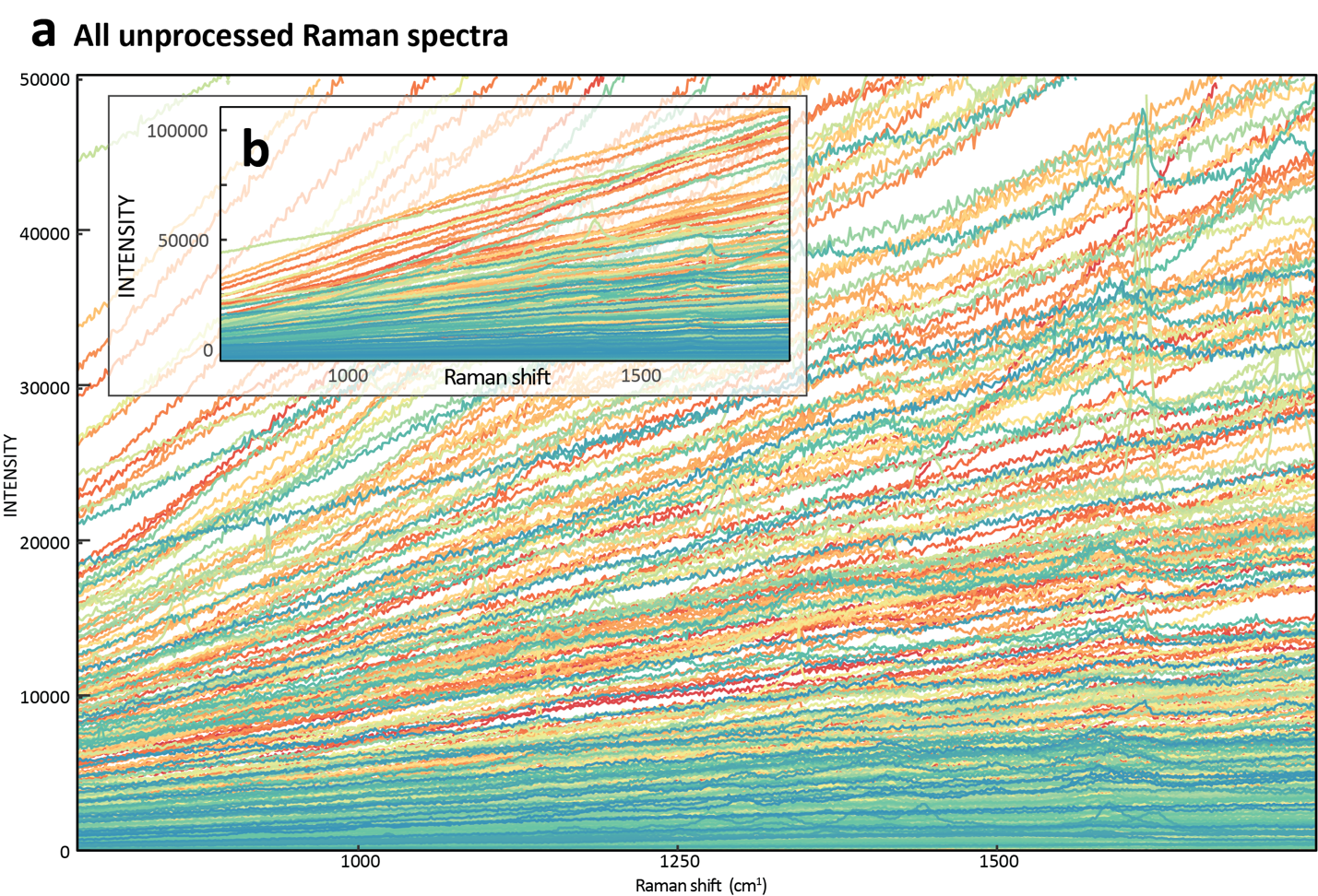


**Figure S5. Plot of all processed spectra including water samples, reference material, fishing gear, and biological samples.** This plot highlights how many of the samples sourced from the non-reference materials (n = 428) exhibited elevated readings of fluorescence in the spectra. Fluorescence was remedied via baseline correction. **a**) Raman spectra intensity values (y-axis) from 0 to 50000 to better visualize the presence of peaks in samples. **b**) a fully zoomed-out y-axis used to highlight the large volume of samples with elevated fluorescence that was corrected for in the preprocessing described in the methods.


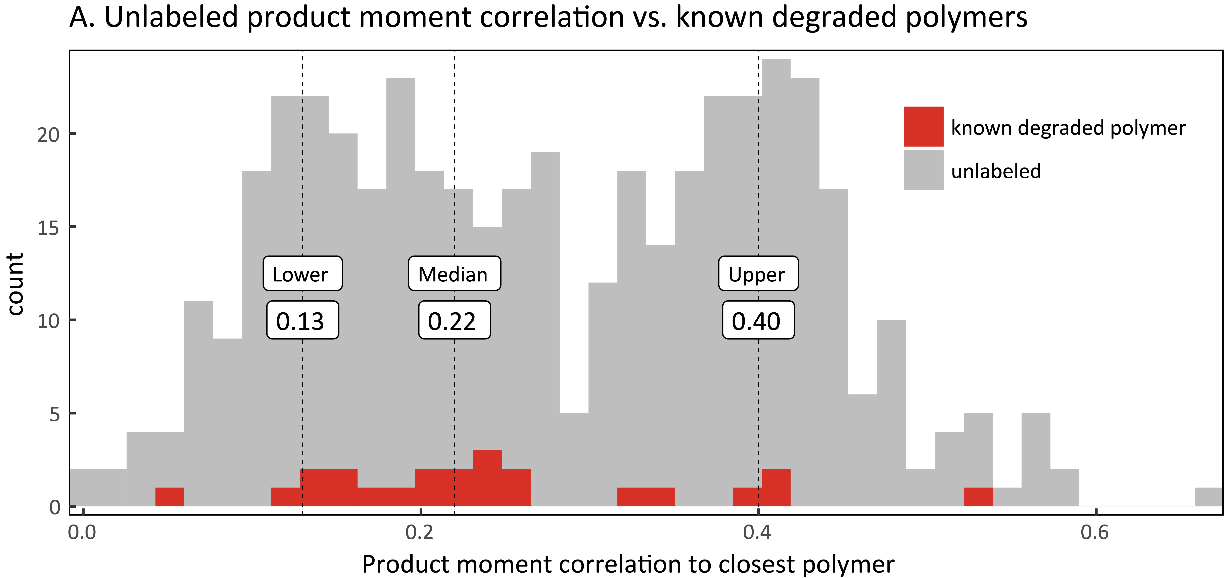


**Figure S6. Establishing cutoff values with known polymer similarity measures against unlabeled specimens.** The red histogram is the distribution of product moment correlation values for known polymer spectra relative unlabeled spectra (gray). This distribution was used to establish cutoff values for identifying plastics based on the observed similarity measures for known plastics. Lower, Median, and Upper represents the 5%, 50% and 95% quantiles.


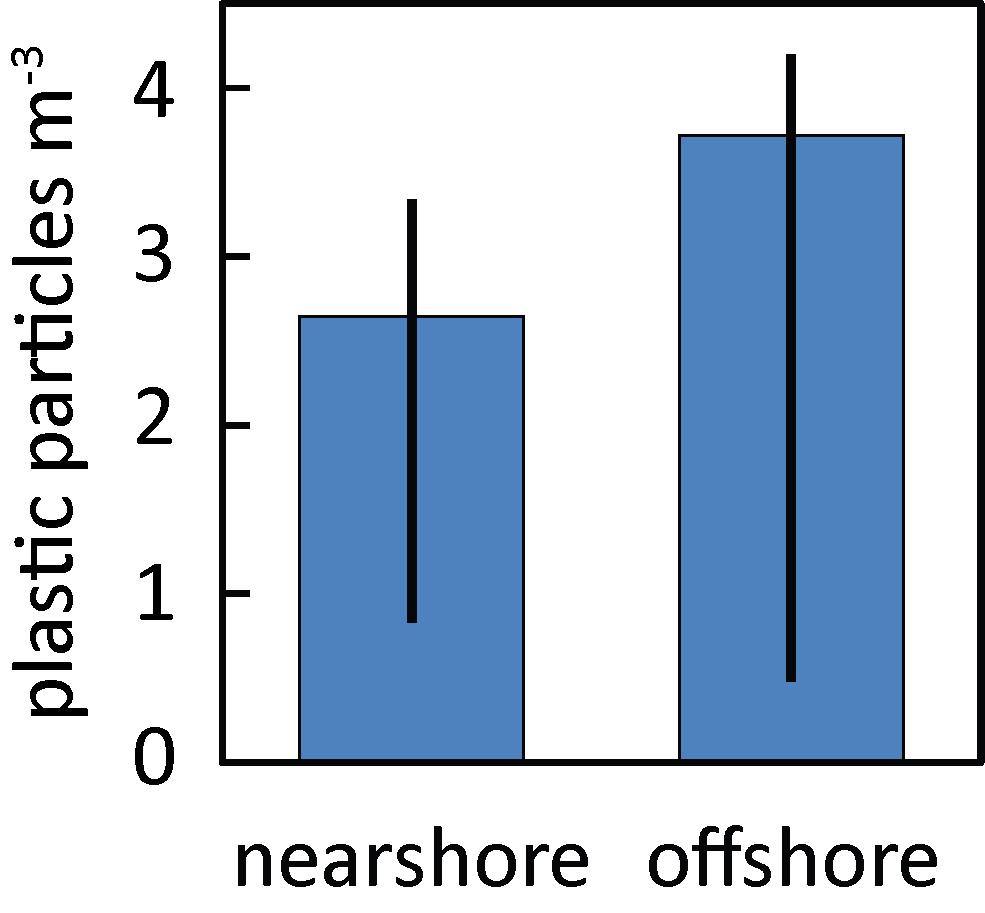


**Figure S7. Microplastic concentrations in the water column, at nearshore and offshore sites in Monterey Bay, California.** Concentrations are averaged across the same sampled depths (5, 25, and 50 m depths) at both onshore and offshore sites (see Fig. S2). Offshore samples were higher in concentration, suggesting that the California Current may supply more plastic than land-based sources in Monterey Bay (see Fig. S2), however, further research is required to understand these differences in more detail Error bars indicate the 5% and 95% quantiles from the Pearson distances (see Figure S4 for more information). Sample sizes were not sufficient to compare plastic concentrations between the onshore and offshore sites. However, across the onshore and offshore shared depths (5, 25, and 50 m), the highest concentration was 11 plastic particles/m^3^ at 25 m depth at the onshore site. High variability was observed for replicate samples taken onshore at 25 m depth (4 and 11 plastic particles/m^3^). Variability between replicate samples at the offshore site was low (25 m: 8 and 9 plastic particles/m^3^; 100 vs. 112.5 m: 9 and 9 plastic particles/m^3^, respectively).


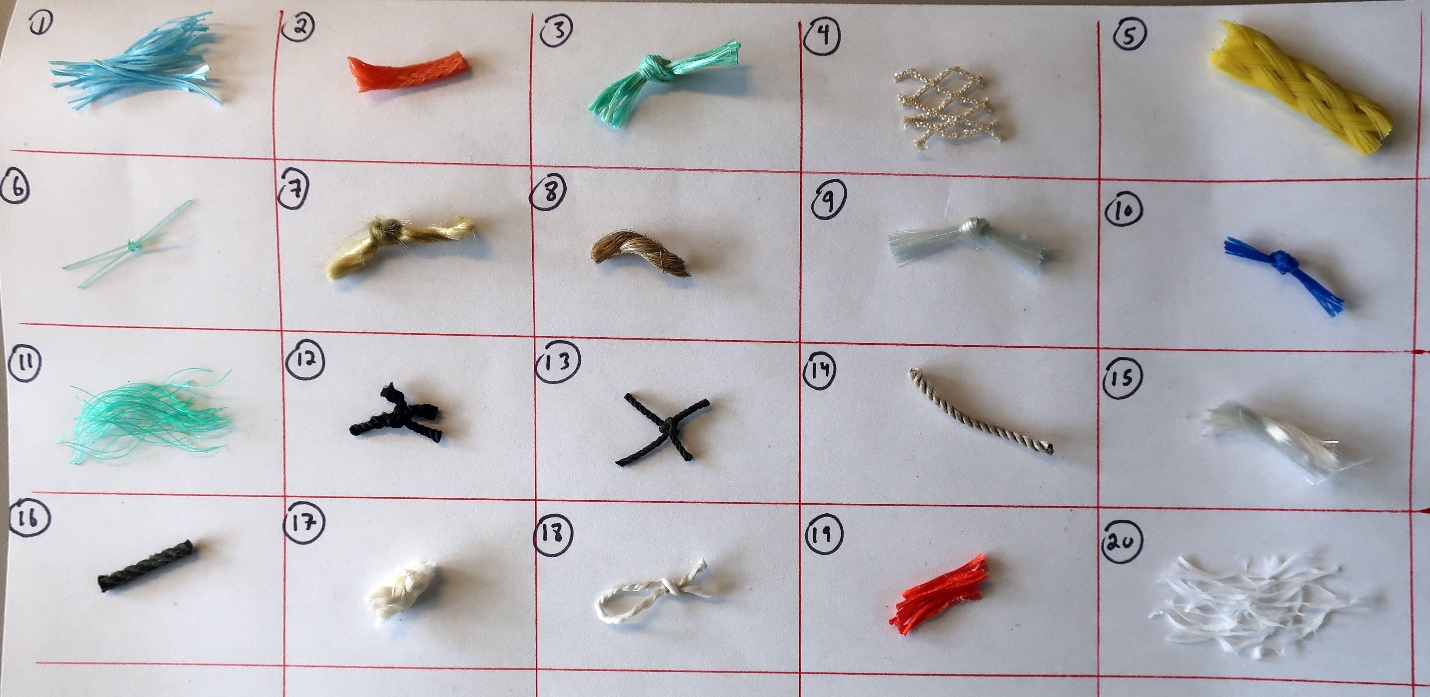


**Figure S8. Maritime polymer specimens representing boat and fishery operations in Monterey Bay, California.** These materials were used in fishery and boat operations and therefore exposed to environmental weathering and degraded from their original industrial form. The materials were subsequently analyzed in a laboratory by laser Raman spectroscopy, and their spectra compared to the spectra from pristine, type-verified specimens from industrial sources. A full description of these materials is provided in Table S1.

**Table S1. Descriptions and applications of 20 maritime polymer specimens used to calibrate Raman spectra distances.** We selected a variety of types of plastic used in Monterey Bay fisheries and boat operations. Selection was based on renderings, colors, forms, braids, and known uses in order to represent a broad diversity of operations. “Chart No.” refers to the image position in Figure S8. “Material assignment” is the identified polymer as determined by the most similar Raman spectra polymer, and “Pearson Distance” is derived from the product moment correlations (where “1” is an exact match). “Material / Gear Description” is the details on the specimen physical form and common renderings (i.e. in nets, lines, etc.). “Fishery application” is the fisheries or species groups in which the materials are commonly employed.

Specimen #15 was run twice as it was perceived to be composed of two materials of varying densities. Specimen #7 was not run, but with specimen #17 was part of a single braided rope and perceived to be of the same material. Specimens #3 and #11 were disintegrated versions of the same material rope, and given both their assignment (PSA) and their Pearson distances (0.211, 0.237) were similar, this provides empirical support for the rigor of our methods. All specimens were actively used in fishery operations, except #9 and #20. Specimen #9 was used in boat repair and #20 was part of the experimental ROV sampler (Fig 1A, Fig S3). All full descriptions of the polymers analyzed in our reference library are available in Table S2.

**Table S2. The 14 plastic polymers included in our reference library of Raman Spectra, alongside their primary material applications.** For the first 12 polymers, we obtained pristine samples from commercial industrial vendors, and used laser Raman spectroscopy to generate diagnostic reference spectra of Raman shift (see Fig, S9). We initially included nylon (N) on this list (making a reference library of 13 total polymers) but were repeatedly unable to obtain usable spectra due to inconsistencies in spectra intensity readings during acquisition of reference samples scans from our samples. However, as N is a notably similar material with a similar spectral output to polyamide (PA), we used PA as a proxy spectrum for N.

Though open-source laser Raman spectra are not widely available, we obtained additional spectra for POM and PMMA from a published study (*3*). To foster broader use in open-source analytical frameworks, the Open Science Framework repository associated with this paper (available at osf.io/j6gmx/) provides the full Raman spectra (range 780-1750 cm^-1^) for all the above materials in non-proprietary (*.csv) formats. “Code” are the polymer name abbreviations used throughout the main text and figures. “Specific gravity” is the density relative to water, where values <1 indicate material is positively buoyant in fresh water (<1.025 for sea water). “Material application” are common uses of each polymer, and “Spectra source” is the origin of the reference Raman spectra. Material applications and specific gravities are adopted from (*12, 13*).


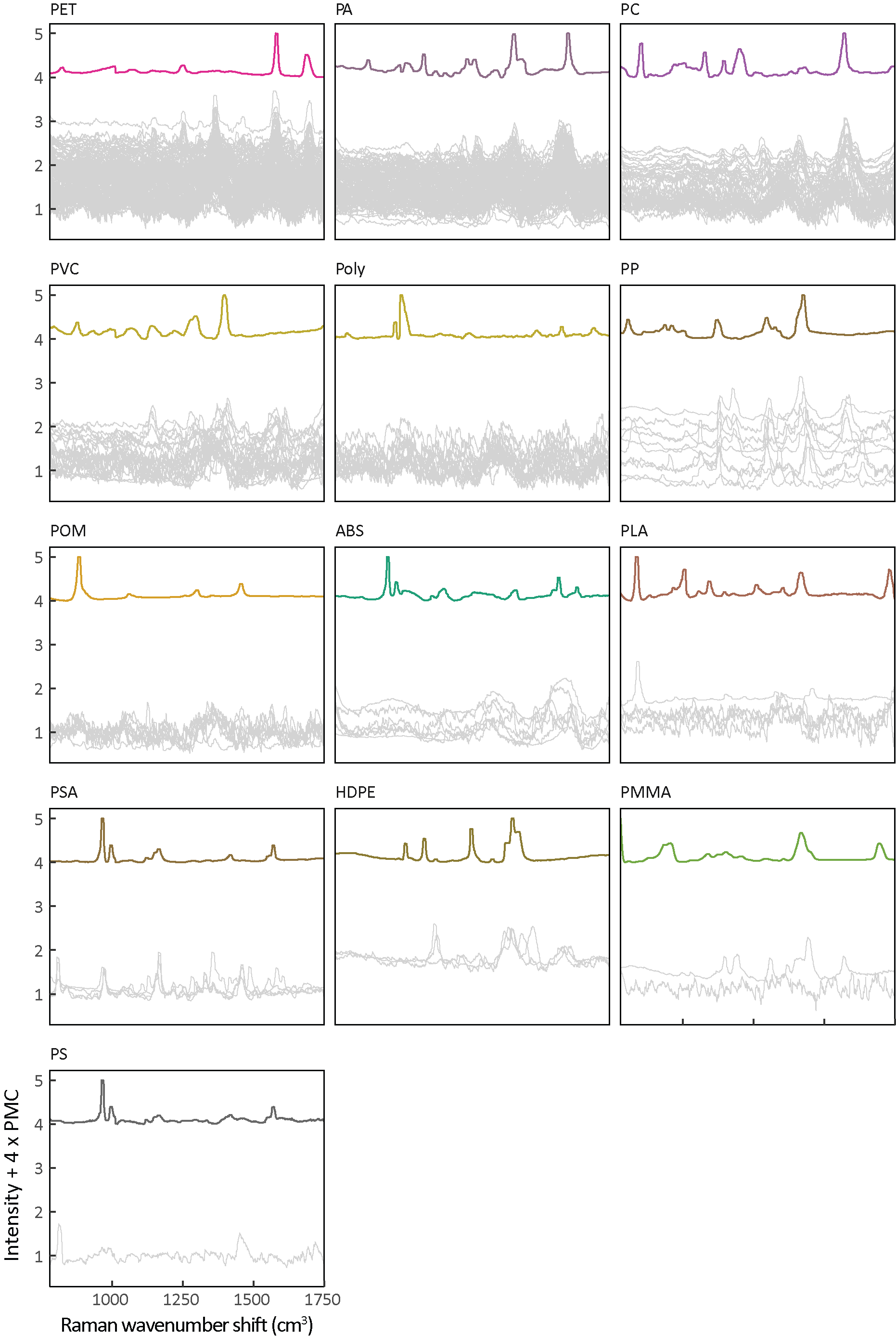


**Figure S9. Spectra of polymer assignments of unlabeled Raman spectra.** The top spectrum of each panel is the labeled reference polymer. All gray lines represent the spectra of particles assigned to that polymer post lower-limit cutoff (0.13). The intercept of the spectra is adjusted to highlight spectra closer the reference spectra were detected as most similar, whereas the furthest away are least similar, though still above the lower limit cutoff for assignment. While in our reference library of spectra (see Table S2), none of the microplastic samples were identified as LDPE.


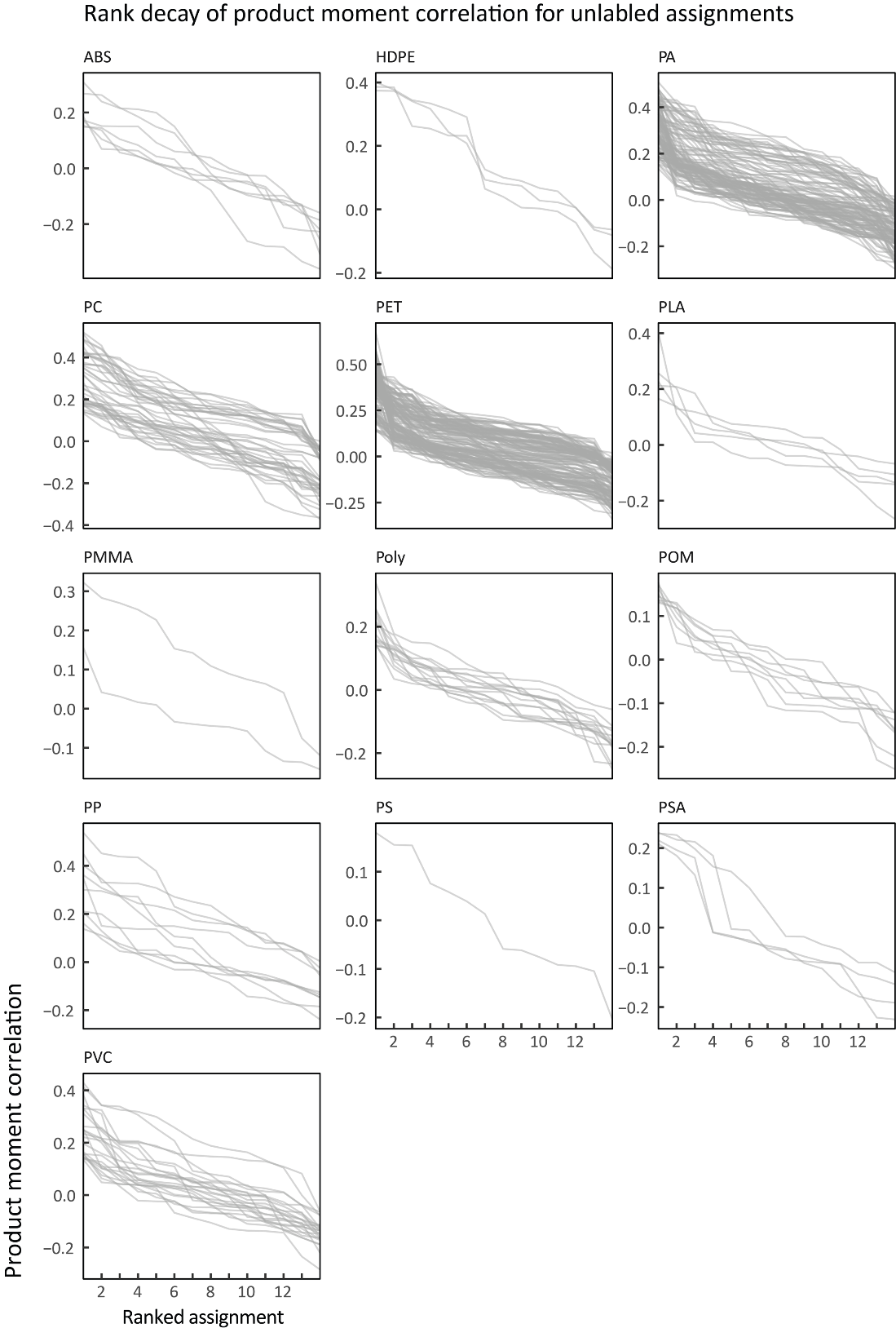


**Figure S10. Ranking of polymer assignments relative and subsequent measure of similarity.** Steeper declines from rank 1 to 2 suggest more confidence in the assignment of unlabeled spectra. For example, PA, POLY, and PET assignments appear to suggest more confidence than POM, ABS, or PSA. Though commonest materials identified, particles identified as PET and PA also had the greatest similarities to the reference spectra. This may indicate that these materials are less degraded in the ocean, either as a result of less exposure (younger materials with less time at sea) or more material durability.


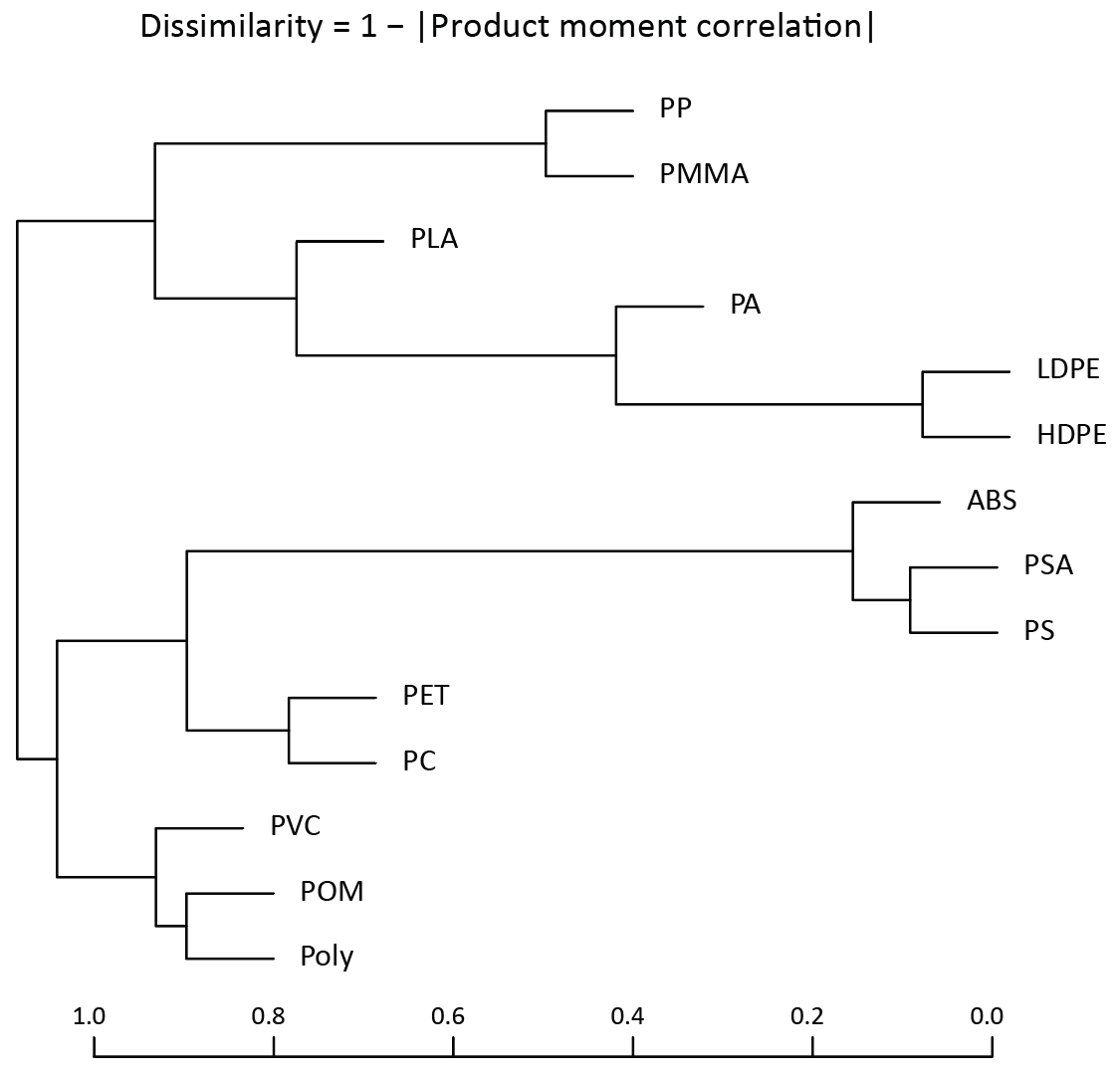


**Figure S11. Hierarchy of spectral similarity of the 14 plastic polymers referenced in this study.** This is the dendrogram from a hierarchal clustering of the correlation matrix for the reference library of polymer spectra. This represents clustering tendencies of Raman spectra given their pairwise product moment correlation. We can see corroboration of the spectral similarity that this characterizes in the clear localization on the dendrogram of HDPE and LDPE due to the high correlation of their spectra.


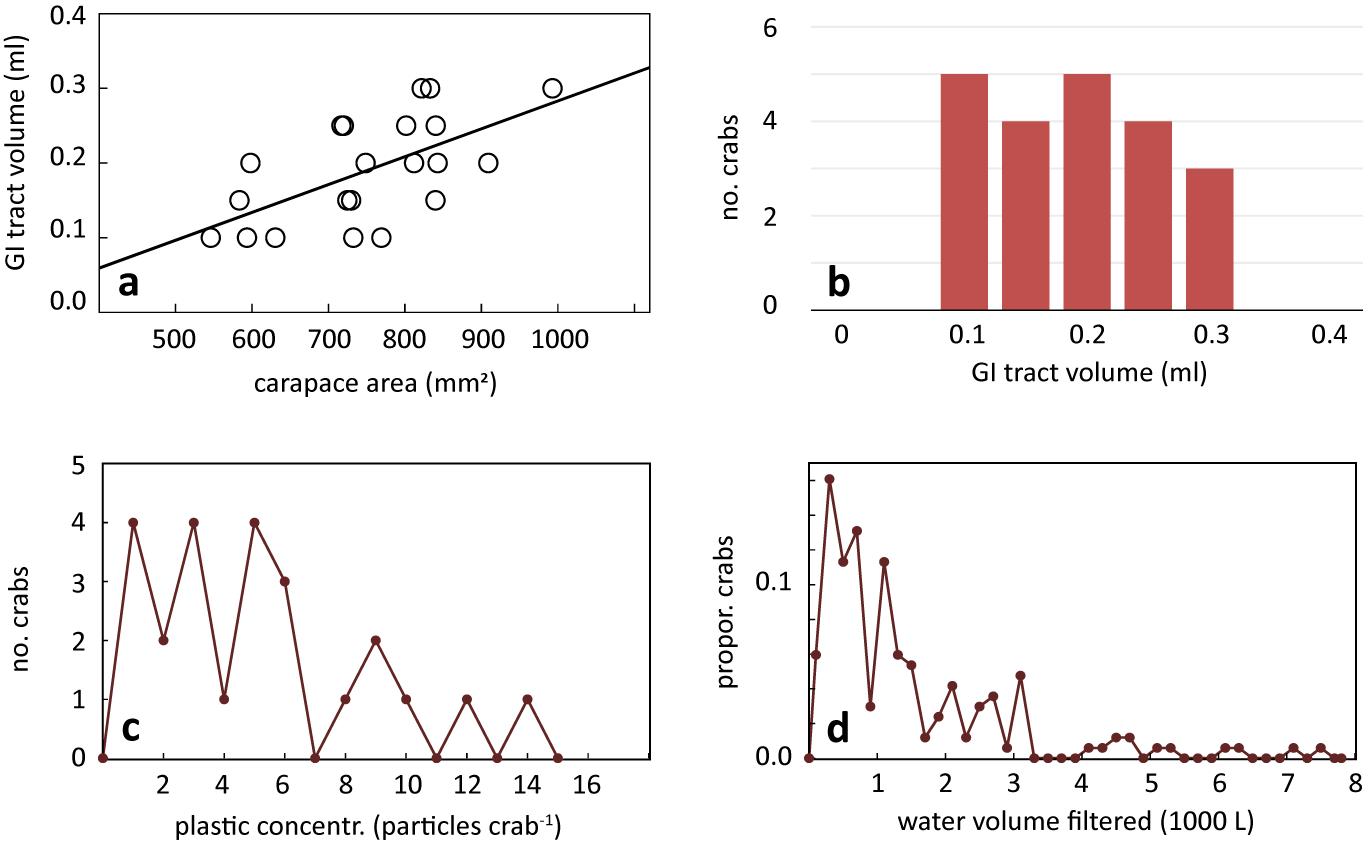


**Figure S12. Microplastic particle counts in pelagic red crabs (*Pleuroncodes planipes*) and the water volumes likely searched to obtain those particles.** a) Basic morphometric relationship of pelagic red crab carapace area (dorsal length x dorsal width) and gastrointestinal (GI) tract volume. Entire GI tracts were removed via dissection and submerged in a partially-filled graduated cylinder. The subsequently displaced water volume indicated the GI tract volume. Crab samples used to derive this relationship were not part of the microplastic analysis. However, the empirical area-to-volume relationship here was used to calculate b) estimates of the volumes of the crab specimens used in this study, from the carapace measurements made prior to their use in the microplastic analyses. c) The distribution of raw numbers of plastic particles found in the GI tract of individual pelagic red crab samples (*n* = 24). Though roughly half of the samples (*n* = 11) have fewer than 5 particles per individual, 3 individual samples had ≥ 10 plastic particles in their GI tract. d) Estimated water volume search rates that pelagic red crabs may have exercised in order to obtain the observed amounts of microplastic particles found in their GI tracts (median = 1,015 L). This calculation is determined from the empirical observations of plastic particles in the GI tract (panel c), known depth preferences of pelagic red crabs (from 0-200m, see main text, Fig. 3A), the plastic particle concentrations at those depths (main text, Fig. 1B), and conservative estimates from Monterey Bay Aquarium aquarists that GI tract passage time is complete for all ingested items at ~24 hours. To account for the variability we observed in plastic concentrations, (d) presents the calculated encounter rates for each and all sampled depths from 0-200m.


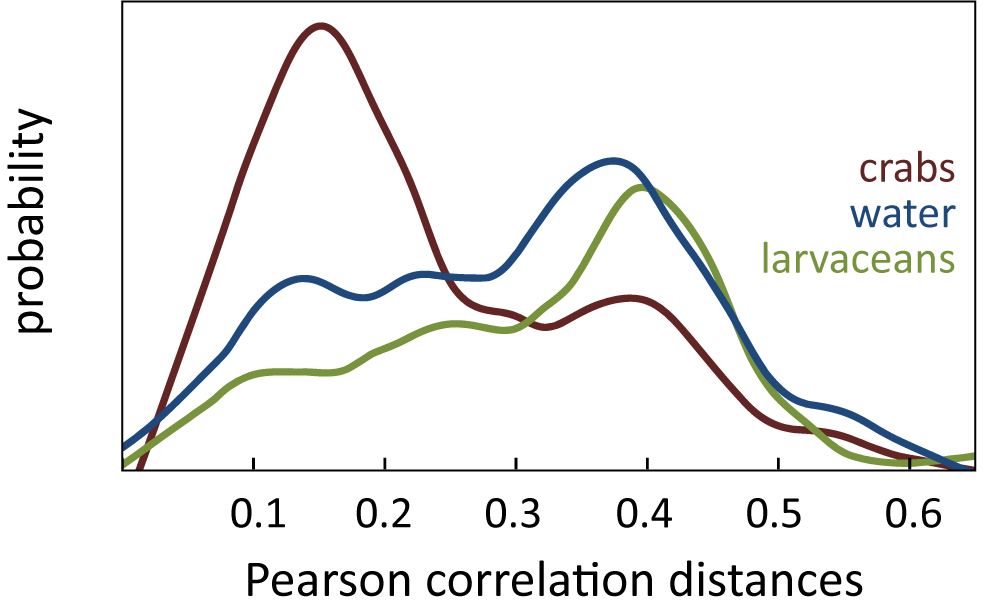


**Figure S13. The plastic found within pelagic red crabs was more degraded than microplastic particles from the water column or found within larvacean sinkers.** Empirical distributions of the Pearson product moment correlations for the plastic particles found within the three sample categories: water, giant larvaceans, and pelagic red crabs. Pearson distances are the product moment correlation coefficients between plastic particle spectra and the nearest match in the polymer reference library. As our reference library contains the most commonly sampled marine plastic types, this measure may indicate increased degradation in pelagic red crabs. Unlike the water and larvaceans samples, the crab samples were taken from the gastrointestinal tract, and were in the process of digestion. Future research of this nature may offer insights into the ability of marine life to break down microplastic through digestive action.


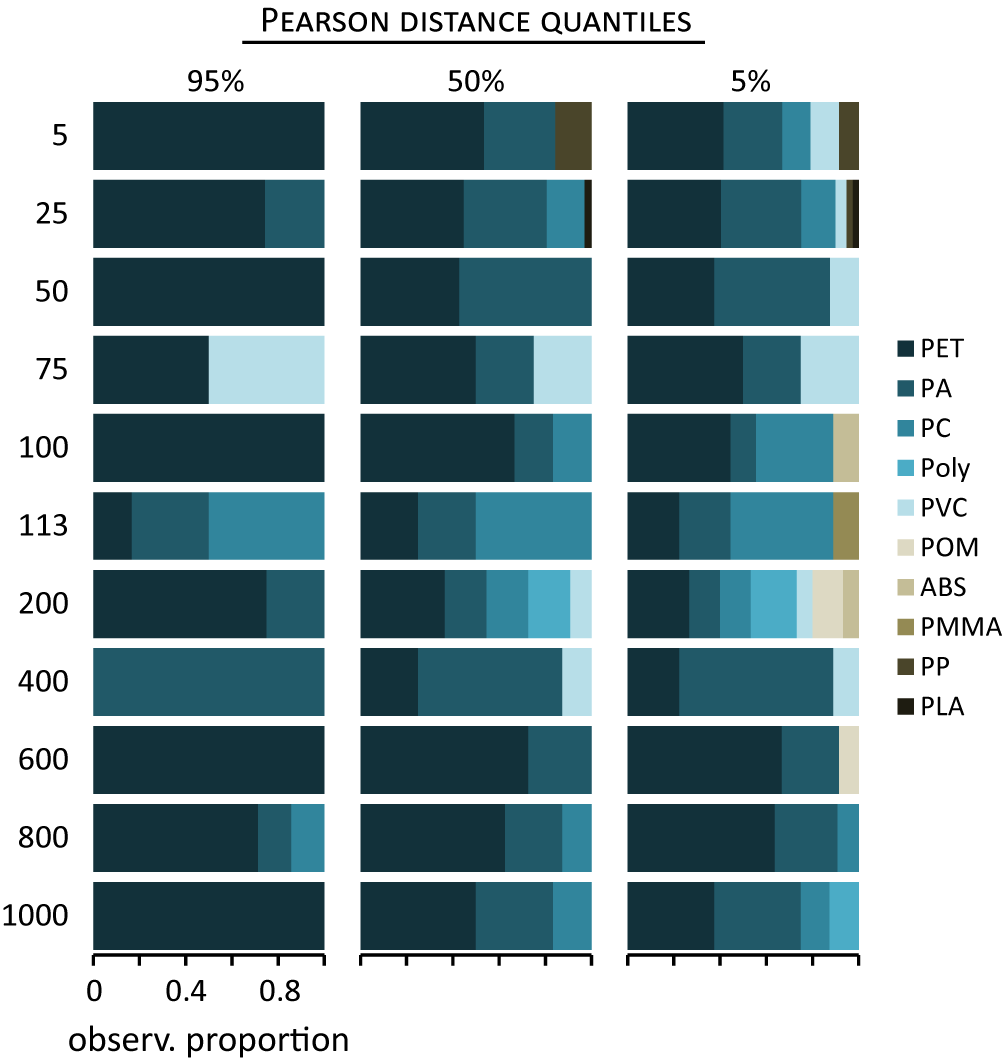


**Figure S14. Polymer types of filtered water samples at varying depths.** Distribution and suspension of polymer type by depth is likely a factor of polymer density, water temperature, salinity, and the stochastic nature of physical processes influencing water column mixing. Results vary depending upon the Pearson distance cutoff, here shown as the 5, 50, and 95% values (0.13, 0.22, and 0.40, respectively) from the empirical distribution of values from the degraded fishing gear sample set (see Fig. S8, Table S1). Continued sampling will provide more extensive insight into the nature of depth composition of polymers. Low density PP (see Table S2) common in the fishery gear we collected (see Fig. 2) was distributed at shallow depths where fisheries operate and also perhaps due to its relatively relative buoyancy. Fig. 1 shows the concentrations of plastic particles by depth.


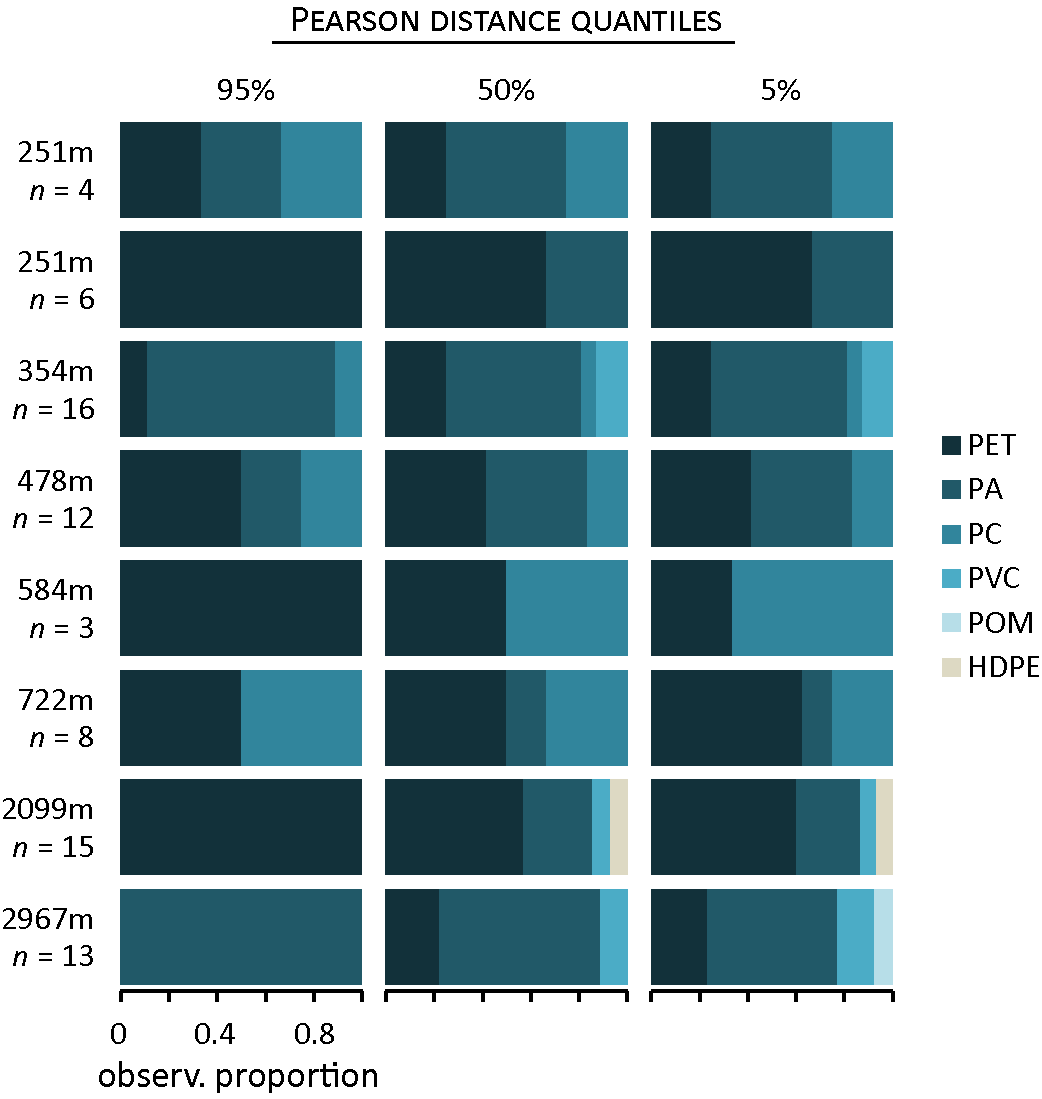


**Figure S15. Polymer types within sampled individual larvacean sinkers at varying depths.** Each row represents the composition of one unique larvacean sinker house, labelled by the depth of collection and the number of plastic particles identified at the Pearson cutoff of 0.13 (e.g., 5% CI). As in Fig. S1 and Fig. S14, composition varies depending on the selected Pearson cutoff for correlation distance to the most similar reference polymer. The two deepest sinkers, and the two immediately below the highest concentration in the water column (see Fig. 1) had the most plastic particles detected, corroborating the idea that sinkers intercept plastics on their descent. Compositionally, most of the sinker samples reflect plastic particles in the water column, at their core habitat depths (Fig. 3C, 200-400 m). HDPE, which was not found in our water samples (Fig. 2, Fig. S14), appeared in one larvacean sinker sample collected at 2,099 m depth. Again, PET and PA are ubiquitous throughout the water column depths.


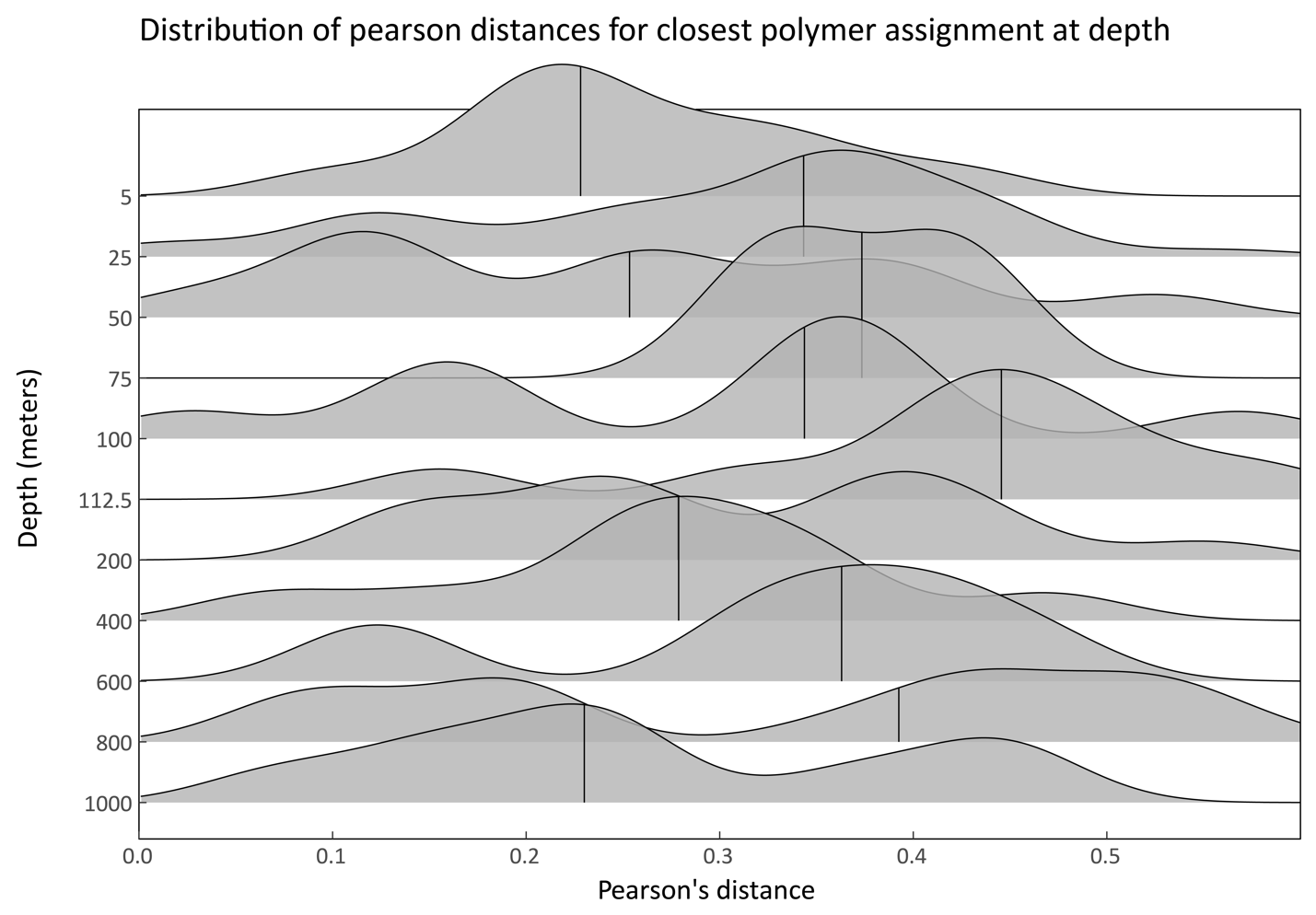


**Figure S16. Pearson distances between microplastics and the closest polymer assignment across sampled depths.** Both the surface and the deep water column contain polymer fibers with the lowest Pearson distances, indicating the greatest dissimilarity between those samples and the closest matching reference polymers in our total sample set. Smoothed histograms represent the observed frequency of Pearson distances for all microplastics sampled in the Monterey Bay water column at each sampled depth. Solid vertical lines within the density plot show the median. This technique may offer promise into understanding the degradation of ocean microplastics, and the patterns and processes involved. Future research will provide further insights and indicate a role of Raman spectroscopy such approaches.

**References**

1. B. H. Robison, The Coevolution of Undersea Vehicles and Deep-Sea Research. *Marine Technology Society Journal* **33**, 65-73 (1999).

2. B. H. Robison, K. R. Reisenbichler, R. E. Sherlock, Giant Larvacean Houses: Rapid Carbon Transport to the Deep Sea Floor. *Science* **308**, 1609-1611 (2005).

3. M. G. J. Löder, M. Kuczera, S. Mintenig, C. Lorenz, G. Gerdts, Focal plane array detector-based micro-Fourier-transform infrared imaging for the analysis of microplastics in environmental samples. *Environmental Chemistry* **12**, 563-581 (2015).

4. C. F. Araujo, M. M. Nolasco, A. M. Ribeiro, P. J. Ribeiro-Claro, Identification of microplastics using Raman spectroscopy: latest developments and future prospects. *Water Research*, (2018).

5. U. B. Cappel, I. M. Bell, L. K. Pickard, Removing cosmic ray features from Raman map data by a refined nearest neighbor comparison method as a precursor for chemometric analysis. *Applied Spectroscopy* **64**, 195-200 (2010).

6. C. A. Lieber, A. Mahadevan-Jansen, Automated method for subtraction of fluorescence from biological Raman spectra. *Applied Spectroscopy* **57**, 1363-1367 (2003).

7. P. Heraud, B. R. Wood, J. Beardall, D. McNaughton, Effects of pre‐processing of Raman spectra on in vivo classification of nutrient status of microalgal cells. *Journal of Chemometrics* **20**, 193-197 (2006).

8. C. Beleites, V. Sergo, hyperSpec: a package to handle hyperspectral data sets in R. *Rpackage version 0.98-20120224, J. Stat. Software,* [*http://hyperspec*](http://hyperspec)*. r-forge. r-project. org, in preparation*, (2012).

9. A. Stevens, L. Ramirez–Lopez, An introduction to the prospectr package. *R Package Vignette, Report No.: R Package Version 0.1* **3**, (2014).

10. R. de Gelder, R. Wehrens, J. A. Hageman, A generalized expression for the similarity of spectra: application to powder diffraction pattern classification. *Journal of Computational Chemistry* **22**, 273-289 (2001).

11. P. H. R. Ng, S. Walker, M. Tahtouh, B. Reedy, Detection of illicit substances in fingerprints by infrared spectral imaging. *Analytical and Bioanalytical Chemistry* **394**, 2039-2048 (2009).

12. J. P. da Costa, A. C. Duarte, T. A. P. Rocha-Santos, in *Comprehensive Analytical Chemistry,* T. A. P. Rocha-Santos, A. C. Duarte, Eds. (Elsevier, 2017), vol. 75, pp. 1-24.

13. S. C. Dexter, *Handbook of Oceanographic Engineering Materials*. (Wiley, New York, ed. 1 edition, 1979), pp. 314.
